# Higher Reliance on Glycolysis Limits Glycolytic Responsiveness in Degenerating Glaucomatous Optic Nerve

**DOI:** 10.1007/s12035-019-1576-4

**Published:** 2019-04-13

**Authors:** Assraa Hassan Jassim, Lucy Coughlin, Mohammad Harun-Or-Rashid, Patrick T. Kang, Yeong-Renn Chen, Denise M. Inman

**Affiliations:** 1 Department of Pharmaceutical Sciences, Northeast Ohio Medical University, 4209 State Route 44, Rootstown, OH 44272 USA; 2 Department of Integrative Medical Sciences, Northeast Ohio Medical University, Rootstown, OH USA

**Keywords:** Seahorse analyzer, Fluorocitrate, DBA/2J, Glaucoma, Mitochondria, Optic nerve

## Abstract

Metabolic dysfunction accompanies neurodegenerative disease and aging. An important step for therapeutic development is a more sophisticated understanding of the source of metabolic dysfunction, as well as to distinguish disease-associated changes from aging effects. We examined mitochondrial function in ex vivo aging and glaucomatous optic nerve using a novel approach, the Seahorse Analyzer. Optic nerves (ON) from the DBA/2J mouse model of glaucoma and the DBA/2-*Gpnmb*^+^ control strain were isolated, and oxygen consumption rate (OCR) and extracellular acidification rate (ECAR), the discharge of protons from lactate release or byproducts of substrate oxidation, were measured. The glial-specific aconitase inhibitor fluorocitrate was used to limit the contribution of glial mitochondria to OCR and ECAR. We observed significant decreases in maximal respiration, ATP production, and spare capacity with aging. In the presence of fluorocitrate, OCR was higher, with more ATP produced, in glaucoma compared to aged ON. However, glaucoma ON showed lower maximal respiration. In the presence of fluorocitrate and challenged with ATPase inhibition, glaucoma ON was incapable of further upregulation of glycolysis to compensate for the loss of oxidative phosphorylation. Inclusion of 2-deoxyglucose as a substrate during ATPase inhibition indicated a significantly higher proportion of ECAR was derived from TCA cycle substrate oxidation than glycolysis in glaucoma ON. These data indicate that glaucoma axons have limited ability to respond to increased energy demand given their lower maximal respiration and inability to upregulate glycolysis when challenged. The higher ATP output from axonal mitochondria in glaucoma optic nerve compensates for this lack of resiliency but is ultimately inadequate for continued function.

**Electronic supplementary material:**

The online version of this article (10.1007/s12035-019-1576-4) contains supplementary material, which is available to authorized users.

## Introduction

Neurons, astrocytes, and blood vessels form a metabolic unit in the CNS. Glucose, lactate, and other metabolic intermediates obtained from the circulation are provided to neurons primarily through astrocytes, and enable glycolysis or oxidative phosphorylation to generate ATP. Neurotransmitters released from neurons can bind metabotropic glutamate receptors on astrocytes that increase intracellular calcium concentration, leading to generation of prostaglandins from arachidonic acid in astrocytes, and ultimately vasodilation to enable increased glucose uptake [1]. The glutamate-glutamine cycle, the astrocytic uptake of glutamate, and release of glutamine for neuronal uptake, can also provide carbons for the TCA cycle. These interactions not only enable flexibility but also engender dependence among the cells of the metabolic unit. Our understanding of these interactions and how they are altered by aging or disease are essential to our ability to manage neurodegenerative disease.

Evidence is emerging to support the critical role of energy management in axon degeneration observed in neurodegenerative disease, including glaucoma [2]. The glial-specific glucose transporter GLUT1 and the neuronal-specific monocarboxylate transporter MCT2 were significantly decreased in optic nerve prior to glaucoma-related degeneration [3]. Astrocytes compromised in their uptake of glucose paired with axons incapable of transporting lactate for fuel would preclude function unless the axons could obtain glucose directly. Neurons are capable of taking up glucose [4], but whether they can do so in a way that would sustain them under such conditions remains to be determined.

The DBA/2J mouse, a widely used model of glaucoma, undergoes a progressive optic neuropathy that results in asynchronous retinal ganglion cell death commencing between 10 and 12 months of age [5]. Deficits in physiological signaling prior to axon transport loss or axon degeneration was observed in the DBA/2J model of glaucoma [6]. Investigation into the cause of the signaling deficit suggested two sources—the mitochondria or the ways by which mitochondria obtain their energy substrate. We determined that the latter contributes to glaucomatous optic neuropathy by showing that critical glucose and monocarboxylate transporters are decreased prior to optic nerve degeneration [3]. The mitochondria, however, also show signs of compromise. Significantly lower mitochondria volume per volume of axon exists in glaucomatous optic nerve [7]. With intraocular pressure elevation, mitochondria in optic nerve of DBA/2J mice exhibited increased fission [8], and mitochondrial cristae loss [9]. It is likely these fragmented mitochondria have inefficient or dysfunctional oxidative phosphorylation, or increased reactive oxygen species production, potentially compromising the high metabolic demand of axons. In support of this, efforts to alter the energy balance (ketogenic diet, vitamin supplementation) toward greater substrate or cofactor availability to support oxidative phosphorylation have demonstrated significant improvement in retinal ganglion cell survival and function [3, 10]. Hence, there are large gains in neural function made possible with providing mitochondria with energy substrate.

An outstanding question is the nature of the axonal mitochondria deficit in the optic nerve. It is not possible to isolate axonal mitochondria from the optic nerve (ON). Therefore, it is necessary to try to physiologically isolate axonal mitochondria and determine their function in situ. This investigation is the first to analyze mitochondrial function in axons using the Seahorse XFe24 Analyzer. Though not designed for tissue, the Seahorse XFe Analyzer has been used to measure oxygen consumption rate in sections or explants of rat and mouse brain [11, 12] and mouse retina [13]; however, no one has yet utilized it to investigate glaucomatous ON. Therefore, we decided to use this method to analyze individual glaucomatous ONs and answer a fundamental question: Whether and how mitochondrial respiration is altered with glaucoma progression. Given that the ON contains mitochondria from axons and glial cells, we would use the glial-specific aconitase inhibitor fluorocitrate to isolate the contribution of axonal mitochondria. Astrocytes and oligodendrocytes that take up fluorocitrate suspend oxidative phosphorylation due to inhibition of the TCA cycle [14]. These methods have shown us that glaucoma ON is energy hungry, with signs of not only increased glycolysis but also oxidative phosphorylation. Despite significant oxygen consumption, glaucoma ON has low maximal respiration. Critically, the glaucomatous ON loses its capacity for metabolic switching—it cannot respond to F_0_F_1_-ATPase inhibition with increased glycolysis.

## Materials and Methods

### Mice

The DBA/2J (D2) mouse, an inbred strain that develops increased intraocular pressure (IOP) secondary to an iris pigment dispersion disease [15, 16], and its control strain, the DBA/2-*Gpnmb*^*+*^ (D2G), were used for all experiments. Mice were obtained from Jackson Laboratories, Bar Harbor, ME. The D2 is a model of secondary glaucoma that recapitulates key features of pathology observed in human patients with primary open angle glaucoma [5, 15, 17, 18]. The D2G control mice share the D2 background but carry a wildtype allele for the *gpnmb* gene.

D2 and D2G mice at 3, 6, and 10 months of age were used for each of two experiments: one with and one without fluorocitrate treatment. For the non-fluorocitrate experiment, 10, 14, and 40 mice each of the D2 and D2G strains at 3, 6, and 10 months of age, respectively, were used. For the fluorocitrate experiments, 10, 10, and 40 each of the D2 and D2G strains at 3, 6, and 10 months of age, respectively, were used. Mice were bred and housed on a 12 h light/dark cycle with access to standard rodent chow and water ad libitum. All procedures abide by the Statement for the Use of Animals in Ophthalmic and Vision Research and were approved by the Northeast Ohio Medical University Institutional Animal Care Committee.

### Visual Acuity Testing

Visual acuities were established in mice using a forced-choice swim behavioral task consisting of 2 sessions of 10 trials per day over a period of approximately 4 weeks. The visual discrimination task apparatus (Cerebral Mechanics) was developed by Prusky [19, 20]. It uses a vertical sinusoidal grating to detect visual perceptual threshold by tasking mice to swim toward the visual stimulus they have associated with a submerged platform. Mouse visual acuity thresholds were determined by systematically increasing or decreasing the grating until animals consistently (≥ 3 sessions) found the platform without error ≥ 7 out of 10 trials per session while failing the next higher spatial frequency for at least 3 sessions. This threshold was recorded as their visual acuity. D2 mice have performed this task successfully at the full range of age and glaucoma-related pathology [21, 22].

### Anterograde Axonal Transport

Mice were anesthetized with isoflurane (2.5%) 3 days prior to sacrifice and 1.5 μl of cholera toxin-B conjugated to AlexaFluor-488 (CTB-488; ThermoFisher Scientific) was injected into the posterior chamber of the eye. After mice were euthanized with an overdose of 390 mg/mL sodium pentobarbital (Beuthanasia-D), brains were dissected out, post-fixed, cryoprotected, then sectioned. Every sixth section was mounted on slides, coverslipped, and imaged at × 50 magnification using an AxioZoom stereomicroscope (Zeiss). An ImageJ-FIJI (Schindelin et al. 2012) macro, available by request, was used to calculate the percentage of the area of the retinorecipient portion of the superior colliculus (percent area fraction) showing fluorescent labeling from the CTB conjugate compared to background labeling as evident in the deep layers of superior colliculus [23]. Data are presented as percent area fraction.

### Intraocular Pressure

Terminal intraocular pressure measurements were taken prior to intraocular cholera toxin-B injections. Mice were lightly anesthetized using inhaled isoflurane (2.5%) and ten consecutive IOP measurements were taken using a TonoLab (Tiolat Oy, Vantaa, Finland) rebound tonometer.

### Seahorse Bioanalyzer

Oxygen consumption rates were measured in acutely isolated optic nerve using a Seahorse XF24 extracellular flux analyzer (Agilent). This plate-based system measures oxygen and proton release using fiber optic sensors that report oxygen consumption rate as well as extracellular acidification rate, representing measures of oxidative phosphorylation and glycolysis, respectively. Using fresh tissue in the Seahorse XF24 required optimization of media and compound concentrations, described below, as well as 40 additional D2 and D2G mice. All compounds and media were from Sigma-Aldrich unless otherwise specified.

**Fig. 1:**
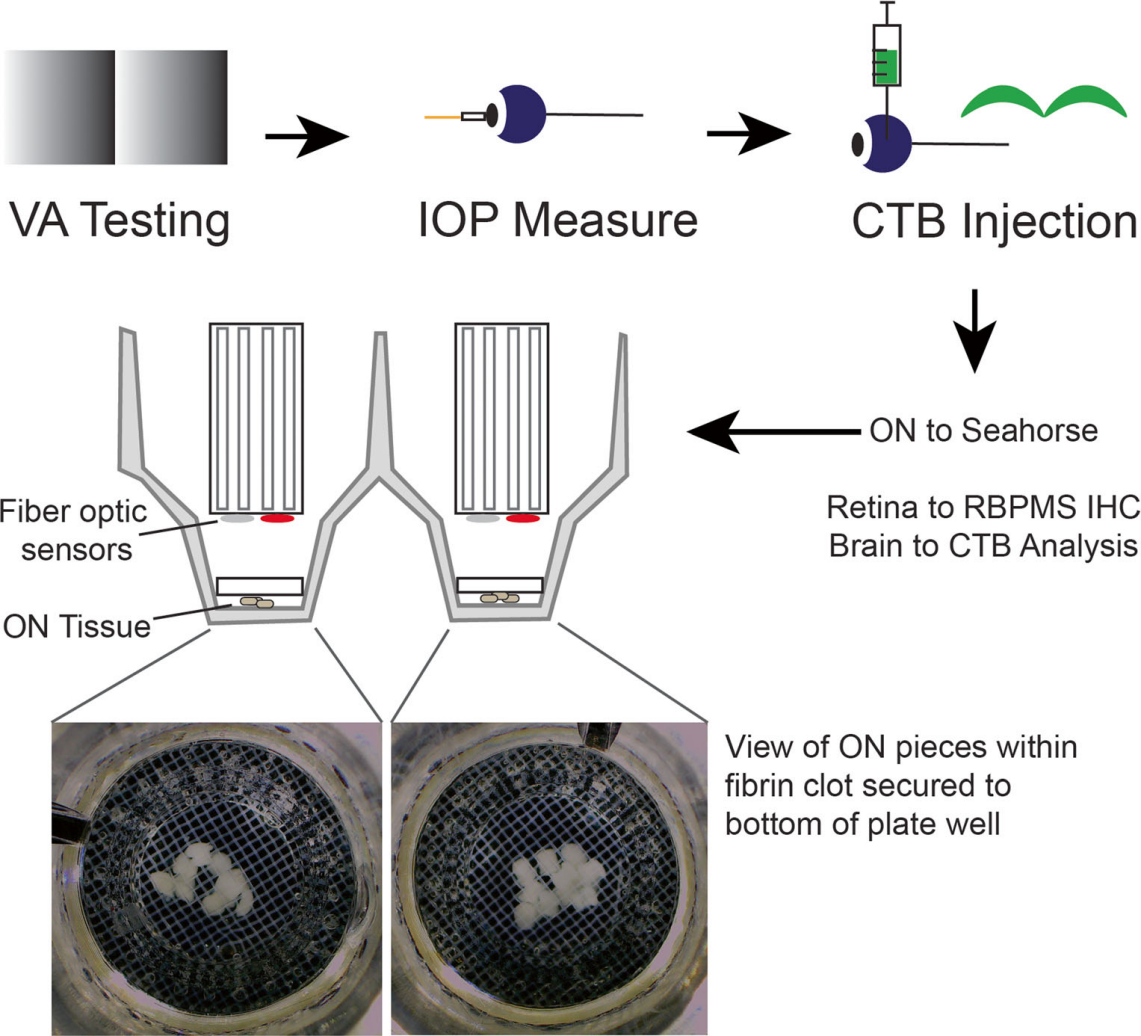
Experimental design. The initial, non-fluorocitrate experiments took place as shown, with mice first placed in the forced-choice swim task in order to establish their visual acuity. Mouse intraocular pressure (IOP) was measured, then mice received bilateral posterior chamber injections of cholera toxin-B conjugated to AlexaFluor-488 (CTB-488). Three days later, mice were sacrificed and optic nerves (ONs) were chopped, secured within a fibrin clot and inverted into a Seahorse Islet Capture plate for analysis. Retinas were dissected, fixed, and immunolabeled with RBPMS. Brains were fixed, sectioned, and analyzed for CTB-488 quantity in the superior colliculus (SC). See “Materials and Methods” for additional detail. The fluorocitrate experiments included IOP measurement and RGC immunolabeling, but did not include CTB-488 injection, nor visual acuity determination

Each Seahorse XF24 Sensor Cartridge plate was incubated in calibrant solution in a 37 °C CO_2_-free incubator 1 day prior to the experiment. Optic nerves (ONs) were carefully dissected from the base of the skull after removing the globe with a scalpel blade, then placed in 37 °C warmed DMEM supplemented with 25 mM glucose, 4 mM glutamine, and 0.5 mM sodium pyruvate (buffering capacity 9.45 × 10^−4^ ± 1.13 × 10^−4^ M). These high substrate concentrations were initially necessary to keep the ONs respiring through the extended assay time. However, for the fluorocitrate experiments, we empirically determined it was feasible to use a DMEM base media with 2 mM glucose, 4 mM glutamine, and 0.5 mM sodium pyruvate (DMEM-low-glucose). Once all ONs were collected for the run, they were removed from DMEM then chopped into 500 μm thick pieces using a McIllwain tissue chopper. Pieces were secured to the mesh insert of a Seahorse XF24 Islet Capture Microplate within a clot made from 5 μL of chicken plasma (Cocalico Biologicals) and 2–5 μL of bovine thrombin. One full ON was placed on each insert (see Fig. 1) then inverted and secured in the microplate well that contained warmed, supplemented DMEM.

Ports were loaded with 75 μL of compounds in solution across the four ports that feed into each well of the sensor cartridge: Port A contained oligomycin A at 10× the final concentration (100 μg/ml); Port B held carbonyl cyanide 4-(trifluoromethoxy)phenylhydrazone (FCCP) (44 μM) and sodium pyruvate (110 mM) at 11× final concentration; Port C was media without metabolic substrates; and Port D held antimycin A at 13× the final concentration (130 μM). In the fluorocitrate experiments, Port A contained fluorocitrate at 10× the final concentration (2500 μM); Port B contained oligomycin A (110 μg/ml) plus either glucose or 2-deoxyglucose (88 mM), both at 11× the final concentration; Port C contained FCCP and sodium pyruvate at 12× final concentration (48 μM and 120 mM, respectively); Port D was loaded with antimycin A at 13× final concentration (130 μM). The sensor cartridge was placed into the Seahorse XF24 to run an automated calibration and equilibration.

The compounds are fed from the ports into each well in sequence (A through D). Oligomycin A inhibits F_1_F_0_-ATP synthase, the enzyme responsible for synthesizing ATP from ADP and a phosphate, thereby decreasing oxygen consumption rate (OCR). FCCP is a mitochondrial uncoupler that allows proton movement across the mitochondrial membrane; its effect is to force the complexes of the electron transport chain to work to maintain the electrochemical gradient, thereby maximizing oxygen consumption. In our preparations, sodium pyruvate was added with the FCCP to further boost mitochondrial respiration. Antimycin A is a complex III inhibitor, the addition of which shuts down mitochondrial respiration. Fluorocitrate is a selective inhibitor of glial cell mitochondrial respiration [24]. Fluorocitrate was prepared as described [25]. Briefly, 8 mg of DL-fluorocitric acid barium salt was added to 1 ml of 0.1 M HCl. Three drops of 0.1 M Na_2_SO_4_ were added to precipitate the salt, followed by 2 ml of Na_2_HPO_4_. The solution was centrifuged at 1000×*g* for 5 min. The supernatant was removed and pH adjusted to 7.4. Fluorocitrate was used at a final concentration of 250 μM [26, 27]. Glucose and 2-deoxyglucose (2-DG) were used as a substrate and non-metabolizable glucose analog, respectively, in the fluorocitrate experiments. OCR values under specific conditions set by injection of each of these compounds can be used to calculate basal respiration, ATP-linked respiration, maximal respiration, spare capacity, and non-mitochondrial respiration. Table 1 shows the calculations behind values expressed in the figures that document OCRs.

**Table 1:** Respiration indices and their means of calculation

Index	Calculation of index value
Basal respiration	Baseline OCR—non-mitochondrial respiration
ATP production	Basal respiration—oligomycin OCR
Maximal respiration	FCCP respiration—non-mitochondrial respiration
Spare capacity	FCCP respiration—basal respiration
Non-mitochondrial respiration	OCR with antimycin A treatment
Coupling efficiency	ATP production/basal respiration

*OCR*, oxygen consumption rate

We undertook several optimization experiments to determine the mix, waiting, and measurement protocols to assess oxygen consumption rate. See Table 2 for the protocol used for experiment 1, without fluorocitrate, and experiment 2, with fluorocitrate. The entire time within-instrument for the ONs was 4 h 45 min.

**Table 2:** Seahorse XF24 protocol

Calibration and equilibration				~ 30 min	Calibrate
					Equilibrate
Baseline				1×	Mix (1.5 min)
				4×	Wait (2.0 min)
					Measure (3.0 min)
Experiment 1: no fluorocitrate	Oligomycin	Experiment 2: fluorocitrate	Fluorocitrate		Inject port A
				1×	Mix (1.5 min)
				3×	Wait (10.0 min)
					Measure (3.0 min)
				3×	Wait (2.0 min)
					Measure (3.0 min)
	FCCP and sodium pyruvate		Oligomycin and 2-DG or glucose		Inject port B
				1×	Mix (1.5 min)
				3×	Wait (10.0 min)
					Measure (3.0 min)
				3×	Wait (2.0 min)
					Measure (3.0 min)
	DMEM w/o Glc, sodium pyruvate		FCCP and sodium pyruvate		Inject port C
				1×	Mix (1.5 min)
				3×	Wait (10.0 min)
					Measure (3.0 min)
				3×	Wait (2.0 min)
					Measure (3.0 min)
	Antimycin A		Antimycin A		Inject port D
				1×	Mix (1.5 min)
				2×	Wait (12.0 min)
					Measure (3.0 min)
				7×	Wait (2.0 min)
					Measure (3.0 min)

### Data Processing

For each baseline OCR, the mean of the last three measurements prior to the first injection was used to represent the baseline OCR. For the oligomycin A response, the minimum OCR value obtained was used, as was the maximal value obtained after FCCP injection. For antimycin A OCR, the mean of the three values after the minimum were used. Wells that did not attain at least a baseline uncorrected OCR of 50 pmol O_2_/min were not used in subsequent analyses; in addition, wells were rejected if the ECAR values were negative.

Following the Seahorse XF24 runs, optic nerves were removed from the mesh inserts and sonicated (2–4 3-s pulses at 10% amplitude) using a Branson Sonifier in 50 μl of protein lysis buffer with protease and phosphatase inhibitors followed by BCA assay (Pierce) to determine total protein concentration. OCR and ECAR data was corrected for the amount of ON protein in each well. OCR data was also normalized to baseline.

For the fluorocitrate experiments, data were normalized to the baseline OCR obtained in the presence of fluorocitrate. Fluorocitrate had a significant effect on OCR, so all subsequent data would be expressed in terms of the fluorocitrate baseline OCR. The data are expressed in graphs as % fluorocitrate OCR.

### Immunohistochemistry

Freshly isolated eyes were immersion fixed in 4% paraformaldehyde for 30 min then cryoprotected in 30% sucrose with 0.02% sodium azide. Retinas were dissected out and vitreous removed. Immunolabeling included washes in 0.1 M PBS, then incubation in blocking solution (5% donkey serum, 1% Triton X-100 in 0.1 M PBS) for 1 h, primary antibody (diluted in 0.5% bovine serum albumin, 0.9% NaCl, 1% Triton X-100 in 0.1 M PBS) for 48–72 h, washes in 0.1 M PBS, blocking solution for 30 min, secondary antibody incubation for 18 h, washes, DAPI labeling, then coverslipping with Fluoromount-G. RBPMS (RNA binding protein, multiple splicing) primary antibody (Genetex, Irvine, CA) to label RGCs (1:250) and secondary antibody from Jackson ImmunoResearch (West Grove, PA) Alexa Fluor 594-conjugated anti-rabbit IgG (1:250) were used. Images were collected on a Leica DMi8 confocal microscope integrated with Leica application Suite X 3.1.1.15751 (Leica Microsystems, Buffalo Grove, IL, USA). Figures were created using Adobe Illustrator, Adobe Creative Cloud version 2017.

### Stereological Analysis

RBPMS-immunolabeled retinal ganglion cells were quantified in whole mounted retinas by unbiased stereology using StereoInvestigator (MBF Bioscience, VT), a × 40 objective, and the optical fractionator approach. Between 35 and 40 sampling sites (10%) of each retina were counted. The coefficient of error (Schmitz-Hof) was maintained at 0.05 or below, ensuring sufficient sampling rate.

### Statistics

Power analysis to determine appropriate sample number was undertaken using GPower 3.1 software. For 80% power and using the standard deviation of OCR, we would need 10 optic nerves per strain per time point. For these experiments, we used at minimum 10 optic nerves, but usually 20 to 40 per time point, especially at 10 months of age. Statistical analyses were performed with GraphPad Prism software version 7.0. Figure graphs were made using GraphPad Prism 7.0. Unpaired, two-tailed *t* tests were used when comparing across groups within a strain, or within a group across strains. For not normally distributed data, non-parametric tests were employed. One-way ANOVA and Tukey’s multiple comparison post hoc test was used when comparing across multiple groups within a strain, or across multiple groups within an outcome measure. *p* < 0.05 was considered significant, and data are reported as mean ± SD.

## Results

**Fig. 2:**
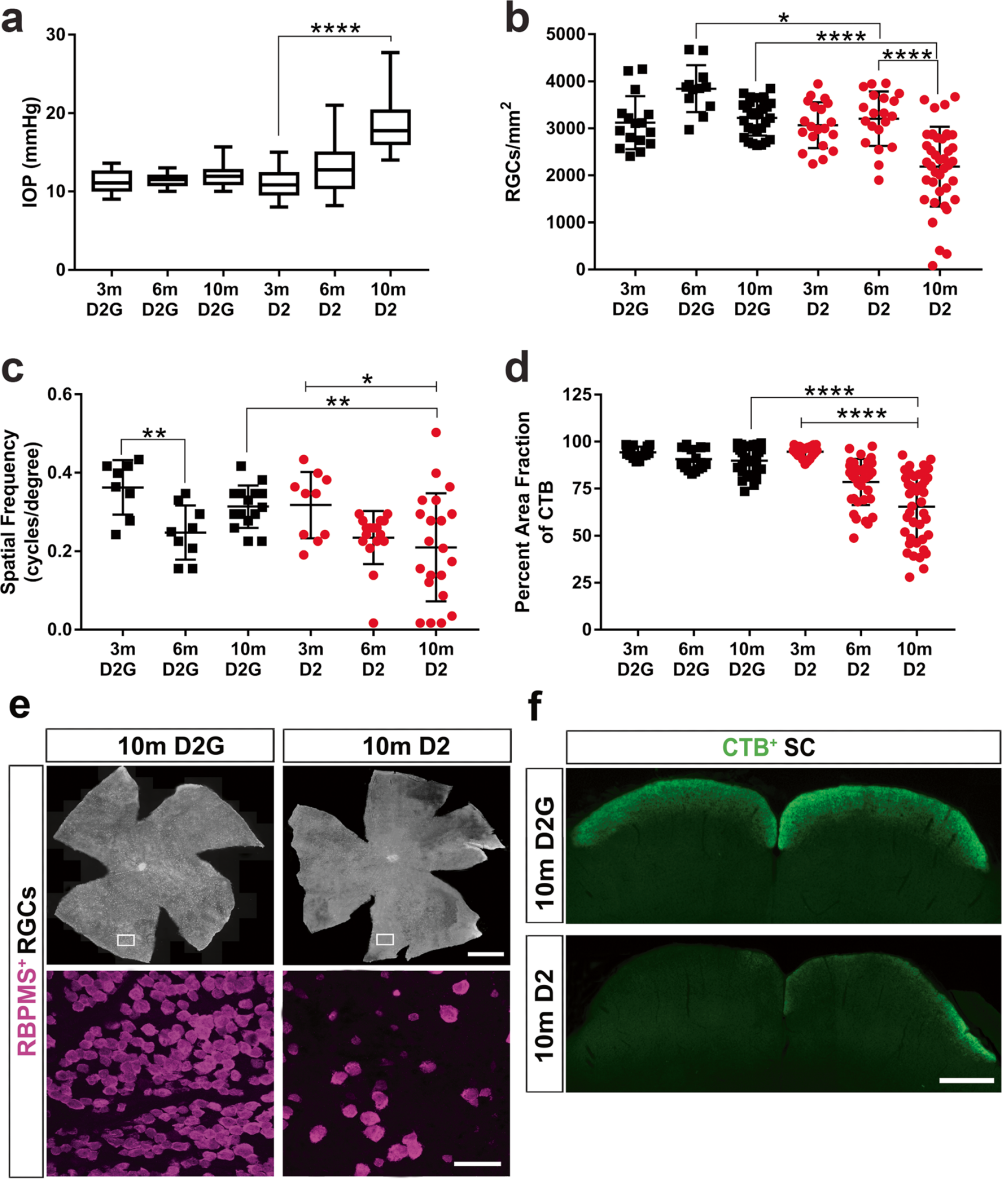
DBA/2J retina and brain show degenerative changes by 10 months of age. **a** Intraocular pressure (IOP) measured in all 3-, 6-, and 10-month-old DBA/2J (D2) and DBA/2J-*Gpnmb*^+^ (D2G) indicates a significant IOP increase by ANOVA (Kruskal-Wallis *H* test, *χ*^2^(2) = 54.19, ****p < 0.0001) in the D2 mice from 3 to 10 months. There was no change in average IOP across the D2G mice. The increases from 3 to 6 months, and 6 to 10 months in the D2 were statistically significant (*t* test, *p* = 0.0091 and *p* < 0.0001, respectively). **b**–**d** Outcome measures for mice in the non-fluorocitrate experiments. **b** Retinal ganglion cell (RGC) density in the D2 and D2G mice at 3, 6, and 10 months of age indicates significantly decreased RGC density in 6 month D2 versus D2G (*t* test, *p = 0.0482) and in 10-month D2 versus D2G (*t* test, ***p = 0.0005). The 10-month-old D2 retina also had significantly lower RGC density than 6-month-old D2 retina (*t* test, ****p < 0.0001). **c** Visual acuity of mice as spatial frequency threshold in cycles/degree shows a significant decline in visual acuity in the D2 mice from 3 to 10 months of age (ANOVA, *F*_2,45_ = 3.482, *p = 0.0393). There was a significant decline in the D2G mice from 3 to 6 months of age (*t* test, **p = 0.0039). The visual acuity was also significantly lower in the 10-month-old D2 versus D2G mice (*t* test, **p = 0.0091). **d** The percent area fraction of cholera toxin-B (CTB) fluorescent label in the superior colliculus (SC) showed a significant decline with aging and pathology in the D2 mice (ANOVA, *F*_2,95_ = 29.95, ****p < 0.0001). There was also a significant difference in CTB label in the 10-month-old D2 versus D2G mice (*t* test, ****p < 0.0001). **e** Photomicrographs of flatmount retina from 10-month-old D2G and D2 mice. Top panels show flatmount retina with white squares to identify the areas from which the high-magnification insets were drawn. Scale bar = 500 μm. Insets show RBPMS-positive RGCs (magenta) in corresponding flatmount retina. Scale bar = 50 μm. **f** Cross-sections of superior colliculus showing CTB labeling (green) in the retinorecipient portions of the superficial layers of D2G and D2 brain. Scale bar = 100 μm

Optic nerves from D2G (control) and D2 (glaucoma) mice were removed and processed as described in Fig. 1. Intraocular pressure (IOP) measured in all D2 and D2G mice at 3, 6, and 10 months of age showed a significant increase in IOP across age in the D2, but no change in the D2G (Fig. 2a). IOP in 10-month-old D2 mice was significantly higher than at 6 months, and IOP at 6 months was significantly higher than at 3 months. IOP increase is accompanied by retinal ganglion cell (RGC) loss in the D2 mice. Figure 2b shows the range of RGC density for retinas taken from the mice used in the non-fluorocitrate experiments. RGCs were immunolabeled with RBPMS, an RGC-specific marker, then quantified using unbiased stereology. There is a significant decrease in RGC number from 6 to 10 months of age in the D2 mice; RGC numbers at 6 and 10 months in the D2 are also significantly lower than RGC density in 6- and 10-month-old D2G mice (Fig. 2b). An expectation of vision loss with RGC loss was observed in the D2 mice. Visual acuity in the mice for the non-fluorocitrate experiments was measured using a forced-choice swim task in which the mice find a hidden platform they have been trained to associate with a visual stimulus [22]. The visual acuity in the D2 mice decreased significantly with aging (Fig. 2c). Visual acuity at 10 months of age in the D2 was significantly lower than that of 10-month-old D2G mice. There was also a significant decline in visual acuity in the D2G from 3 to 6 months of age (Fig. 2c). An additional measure of pathology in the D2 model of glaucoma is anterograde axon transport deficit, as measured in the D2 and D2G mice by quantifying the percent of the superior colliculus (SC) area positive for cholera toxin-B conjugated to AlexaFluor-488 (Fig. 2d). Reduced CTB in the SC indicates a deficit in the RGC transport of CTB from the retina to the retinorecipient regions in the brain [23, 28]. There was a significant decrease in CTB+ area of the SC with age in the D2 mice (Fig. 2d). There was also a significant decrease in the CTB labeling of the 10-month-old D2 versus the 10-month-old D2G SC. Examples of RGC distribution in flatmount retina from D2G and D2 10-month-old mouse eyes are shown in Fig. 2e, with high-magnification insets. Figure 2f depicts a typical CTB labeling result in the D2G and D2 SC.

### Oxygen Consumption Rate in Optic Nerve

**Fig. 3:**
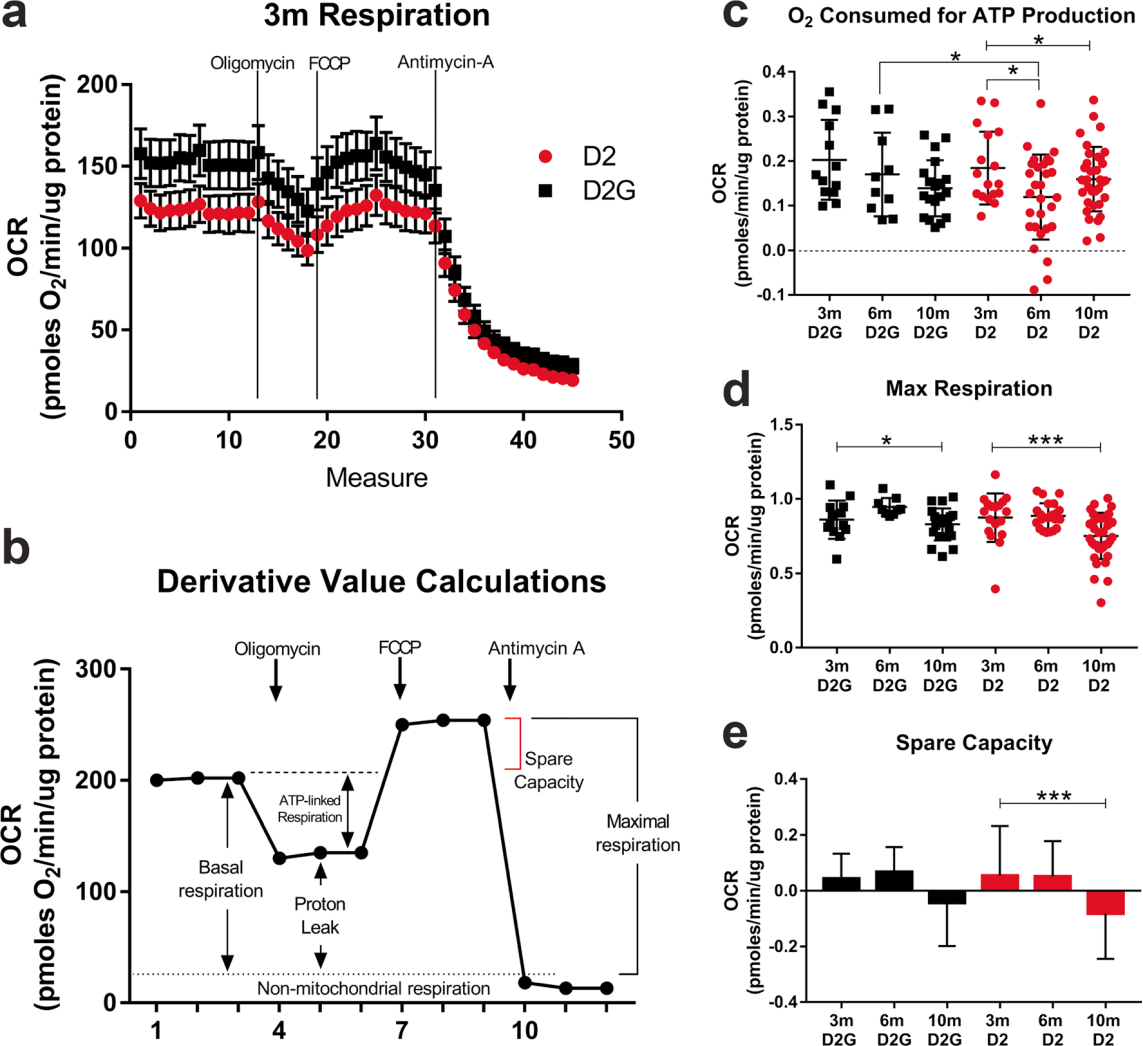
Oxygen consumption rate (OCR) in D2 and D2G ON, normalized to amount of ON protein per well, used to calculate ATP production, maximal respiration, and spare capacity in ON. Media for these experiments contained 25 mM glucose, 4 mM glutamine, and 0.5 mM sodium pyruvate. **a** Mitochondrial respiration in the 3-month-old D2G ON (black squares), and the 3-month-old D2 ON (red circles). There were no statistical differences for OCR taken after oligomycin, FCCP, and antimycin A injections across strain within the 3, 6, and 10-month-old age groups; only the 3-month-old graphs are shown as example output. OCR is pmol O_2_/min/μg protein ± SEM for this graph only, to allow individual points to be visible. **b** Schematic showing the various calculated values for basal respiration (baseline), ATP-linked respiration (often referred to as ATP production), maximal respiration, and spare capacity, as derived from the oxygen consumption rate measured during a Seahorse Analyzer run. Arrows show the injection points for the oligomycin, the FCCP, and the antimycin A compounds that inhibit the F_0_F_1_-ATPase, uncouple the mitochondrial membrane potential from ATP production, and inhibit Complex III of the electron transport chain, respectively. **c** ATP production decreased significantly with age in the D2 ON (ANOVA, *F*_2,78_ = 3.632, *p = 0.031). ATP production was significantly lower in the 6-month-old D2 ON when compared to 3-month D2 (*t* test, *p = 0.0236) and the 6-month-old D2G ON (*t* test, *p = 0.032). ATP production did not vary across ages in the D2G mouse ON. **d** Maximal respiration in the D2 and D2G ON decreased significantly with aging (within strain for D2 Kruskal-Wallis *H* test, *χ*^2^(2) = 14.78, ***p = 0.0006; for D2G, ANOVA, *F*_2,40_ = 3.771, *p = 0.0316). **e** Spare capacity, the potential for acceleration of ATP production when necessary, was significantly decreased with aging in the D2 but not the D2G ON (within strain for D2 Kruskal-Wallis H test, *χ*^2^(2) = 16.8, ***p = 0.0002; for D2G Kruskal-Wallis *H* test, *χ*^2^(2) = 5.597, *p* = 0.0609)

Optic nerves from the mice whose retinas and SC were analyzed as described above were removed and used for the Seahorse XF Analyzer experiments that were designed to evaluate mitochondrial function by measuring oxygen consumption rate through the sequential addition of inhibitors of oxidative phosphorylation. The average oxygen consumption rate (OCR) for 3-month-old D2G and D2 ON is shown in Fig. 3a. These data represent the respiration for all mitochondria within all of the cell types (astrocytes, oligodendrocytes, microglia, endothelial cells) and axons in the ON. Initially, data were corrected for the amount of protein within the well (after the Seahorse run, ONs were removed and concentrations measured) then normalized to baseline OCR. Comparisons of OCR after oligomycin, FCCP, or antimycin A injections were not statistically different within age and across strain (Fig. 3a).

ATP production and other data derived from OCR values are calculated as depicted in Fig. 3b. Oxygen consumed for ATP production did not vary across ages in the D2G mouse ON. In the D2 mouse ON, ATP production decreased significantly with age, Fig. 3c. ATP production was also significantly lower in the 6-month-old D2 ON when compared to 3 month D2 and the 6-month-old D2G ON. The 10 month-old D2 ON oxygen consumption for ATP production did not differ from the 6- or 10-month-old D2G ON. Maximal respiration in the D2 and D2G ON decreased significantly with aging (Fig. 3d). Spare capacity, the potential for the system to accelerate ATP production, was significantly decreased with aging in the D2 but not the D2G ON (Fig. 3e).

### Extracellular Acidification Rate Highest in Glaucoma Optic Nerve

**Fig. 4:**
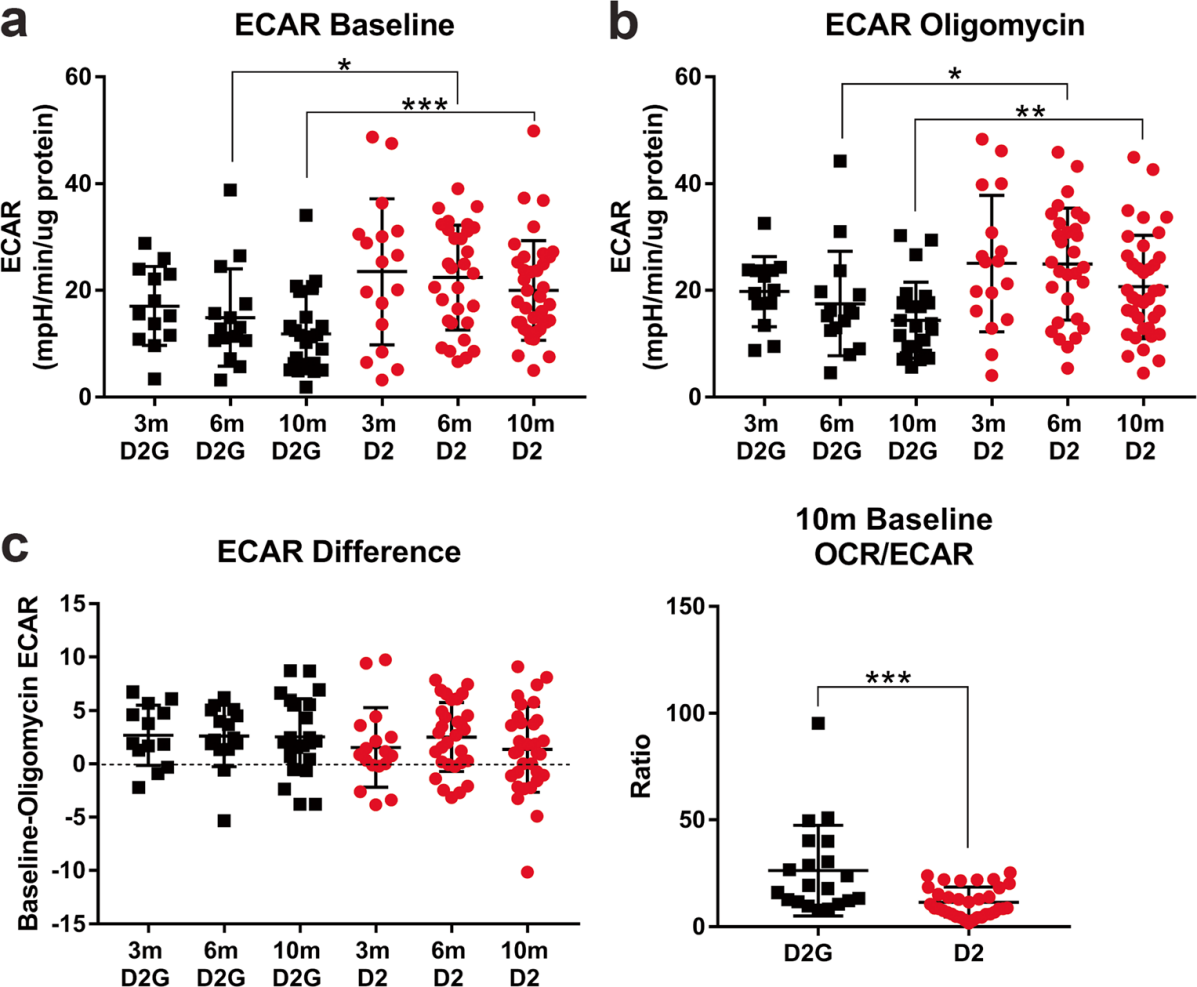
Extracellular acidification rate (ECAR) normalized to protein per well in 3-, 6-, and 10-month-old D2 ON. **a** Baseline ECAR, normalized by amount of protein per well, was not statistically different in the D2 versus the D2G ON at 3 months of age (*t* test, *p* = 0.0564), but was significantly higher in the D2 ON versus the D2G at 6 months of age (*t* test, *p = 0.013). Baseline ECAR was significantly higher in the 10-month-old D2 ON compared to the D2G (*t* test, ***p = 0.0003). **b** ECAR measured in the presence of oligomycin was not significantly different in the D2 versus D2G ON at 3 months of age (*t* test, *p* = 0.1863), but was significantly higher in 6-month-old D2 compared to D2G ON (*t* test, *p = 0.0282). ECAR in the presence of oligomycin was significantly higher in the 10-month-old D2 compared to D2G ON (*t* test, **p = 0.0099). **c** The difference between ECAR at baseline and ECAR in the presence of oligomycin. **d** The ratio of OCR to ECAR in the ONs shows a significantly decreased ratio in the D2 ON (*t* test, ***p = 0.0006)

The extracellular acidification rate (ECAR) represents the release of protons into the extracellular milieu as a byproduct of glycolysis (from lactate production) but also the protons from substrate oxidation that are released with the export of CO_2_ that becomes hydrated to H_2_CO_3_ and dissociates to HCO_3_^−^ + H^+^ [29]. Acidification from respiration compared to glycolysis varies widely by cell type [30]; however, as shown below in the fluorocitrate experiments, roughly half of the ECAR in D2G ON can be attributed to glycolytic activity. From the outset, the ECAR in the D2 ON was higher than in the D2G (Fig. 4a). At 6 and 10 months of age, the D2 ECAR was significantly greater than the D2G (Fig. 4a). These data suggest that glycolysis may provide a larger proportion of energy in the D2 ON than in the D2G, even in spite of the high glucose conditions (see “Materials and Methods”). In the presence of oligomycin, ECAR was significantly greater in the D2 ON compared to the D2G at both 6 months and 10 months of age (Fig. 4b). Since oligomycin inhibits the F_0_F_1_-ATPase, the ECAR measures in the presence of oligomycin may increase over baseline ECAR, reflecting an increase in glycolysis to meet ATP needs. Both the D2 and D2G ON mean ECAR in the presence of oligomycin was increased over baseline ECAR (Fig. 4c), but there were individual ONs for each age and strain that did not show an increase in ECAR with oligomycin treatment.

The conditions of the first experiment supplied excess substrate (0.5 mM aerobic substrate sodium pyruvate and 25 mM aerobic/anaerobic substrate glucose), a result of empirical optimization of conditions that would allow the ONs to respond through the 4+ hours of assay time. The OCR/ECAR ratio indicated the striking preference for glycolysis in the D2 ONs (Fig. 4d). Since the relatively high glucose concentration exceeds the physiological range, we endeavored to execute our fluorocitrate experiments (described below) using physiological glucose conditions (2 mM that rises by 8 mM to 10 mM with FCCP addition).

We also hypothesized that visual function (as measured by visual acuity), RGC number, anterograde axon transport, or IOP levels (Fig. 1) would predict changes in respiration indices. However, we determined this was not the case. For example, a plot of calculated maximal respiration against visual acuity for the D2 and D2G mice showed no difference in the slope of the regression line through the scatterplot, nor did a plot of basal respiration by RGC density (Online Resource Figure 1). ATP production, maximal respiration, or ECAR plotted by visual acuity binned into data ranges also showed no predictive power (Online Resource Figure 2). However, we did note that the higher the visual acuity, the greater oxygen consumption in the presence of FCCP for the D2 ON (Online Resource Figure 2b). Higher visual acuity suggests increased numbers of functional RGCs; these RGC axons show greater capacity for increased oxygen consumption. Finally, the ECAR in the ON taken from mice with visual acuity at 0.2 cyc/deg. or higher was not different across the D2 and D2G strains, but was significantly higher for the D2 mice with low visual acuity (0 to 0.2 cyc/deg), Online Resource Figure 2c. This suggests that mice with low visual acuity drive much of the significantly increased ECAR baseline at 10 months of age as shown in Fig. 4.

### Fluorocitrate Unmasks Axonal Mitochondria Function

**Fig. 5:**
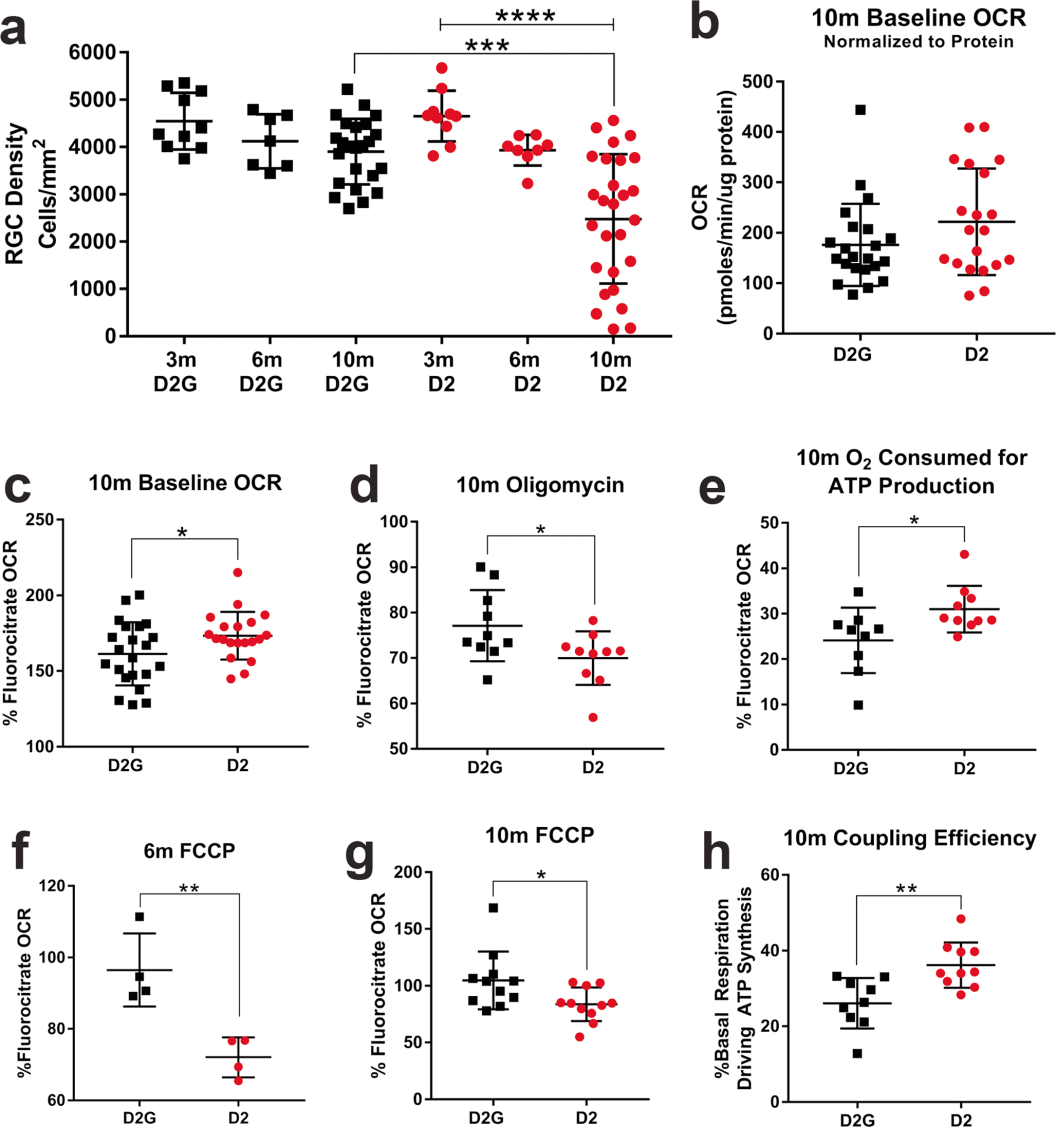
RGC density and mitochondrial respiration for mice used in the fluorocitrate experiments. Media for these experiments contained 2 mM glucose that increased to 8 mM at the oligomycin step, 4 mM glutamine, and 0.5 mM sodium pyruvate. **a**. RGC density in 3-, 6-, and 10-month-old D2 and D2G mouse retina decreased significantly with age in the D2 retina (ANOVA, *F*_2,42_ = 15.96, ****p < 0.0001). The 10-month-old D2 retinas had significantly lower RGC density than age-matched D2G retinas (*t* test, ***p = 0.0006). **b** Baseline oxygen consumption rate (OCR), normalized to protein per well, in the 10-month-old D2G and D2 ON. There was no statistical difference across strain. **c** OCR in 10-month-old D2 and D2G ON with fluorocitrate (FC) treatment indicates significantly higher baseline OCR in the D2 (*t* test, *p = 0.0445). Data are normalized to the OCR with FC treatment and expressed as percent of FC OCR. **d** OCR in 10-month-old D2 and D2G ON in the presence of FC and oligomycin shows significantly lower OCR in the D2 ON compared to the D2G (*t* test, *p = 0.034). **e** ATP production (the difference between oligomycin and FC baseline OCR) is significantly higher in the D2 ON at 10 months of age (*t* test, *p = 0.027). **f** OCR with FCCP treatment in the presence of FC is significantly lower in the D2 compared to D2G ON at 6 months of age (*t* test, **p = 0.0057). **g** OCR after treatment with FCCP, in the presence of FC, shows significantly lower OCR in 10-month D2 ON compared to D2G (*t* test, *p = 0.028). **h** The fraction of basal respiration driving ATP synthesis, or coupling efficiency, is significantly higher in the 10-month D2 ON (*t* test, **p = 0.003)

Fluorocitrate (FC) inhibits the TCA cycle enzyme aconitase in astrocytes because of its preferential uptake by astrocytes, and possibly oligodendrocytes [14, 25]. Incubating optic nerve with FC should better isolate the contribution of axonal mitochondria to the mitochondrial respiration as measured by the Seahorse Analyzer. We injected FC in advance of the other port injections during a subset of Seahorse runs for D2 and D2G ON at 3, 6, and 10 months of age; Online Resource Figure 3c confirms aconitase inhibition with the FC concentration used here. Figure 5a shows the distribution of RGC density for the mice used in the FC experiments. There was a significant decline in RGC number with aging in the D2 retina. RGC number in 10-month-old D2 was significantly lower than in D2G retina (Fig. 5a).

There was no difference in baseline OCR between the D2 and D2G 10-month-old ON when data were normalized to protein (Fig. 5b). However, when baseline OCR was shown as a percentage of the OCR in the presence of FC, the OCR was significantly higher in the 10-month-old D2 than D2G ON (Fig. 5c). This indicated that the glia, whose mitochondria were inhibited with FC treatment, were contributing more to OCR in the D2 than the D2G ON at 10 months of age. Baseline OCR did not differ for D2 and D2G mice at 3 and 6 months of age (data not shown). FC, by blocking glial mitochondria, establishes a baseline oxygen consumption that represents that obtained from axons within the ON; therefore, subsequent data is normalized to the FC baseline OCR. Online Resource Figure 3a and b shows the OCR through each stage of the FC experiment for one example each of the D2 and D2G ON at 10 months of age. Oligomycin injection in the presence of FC resulted in significantly lower OCR in the D2 ON than the D2G (Fig. 5d). The magnitude of oligomycin-associated decrease in OCR reflects ATP-linked respiration, suggesting that D2 axonal mitochondria are producing more ATP than those in the D2G control strain. Figure 5e shows that the oxygen consumed for ATP production (derived from the difference between FC and oligomycin OCR) is significantly higher in D2 than D2G ON in the presence of FC, suggesting the axonal mitochondria in the D2 ON are more productive than those in the D2G ON. Proton leak, the OCR attributable to proton movement across the inner mitochondrial membrane independent of F_0_F_1_-ATPase activity, was not different between the D2 and D2G ON at 10 months (data not shown). In contrast to ATP production, maximal respiration as detected after FCCP injection in the presence of FC was significantly lower in the D2 ON compared to the D2G at 6 months of age (Fig. 5f) and at 10 months (Fig. 5g). Coupling efficiency, the fraction of basal respiration that drives ATP synthesis, is significantly higher in the 10-month D2 ON (Fig. 5h).

### ECAR in the Presence of Fluorocitrate Shows Limits to Glycolysis

**Fig. 6:**
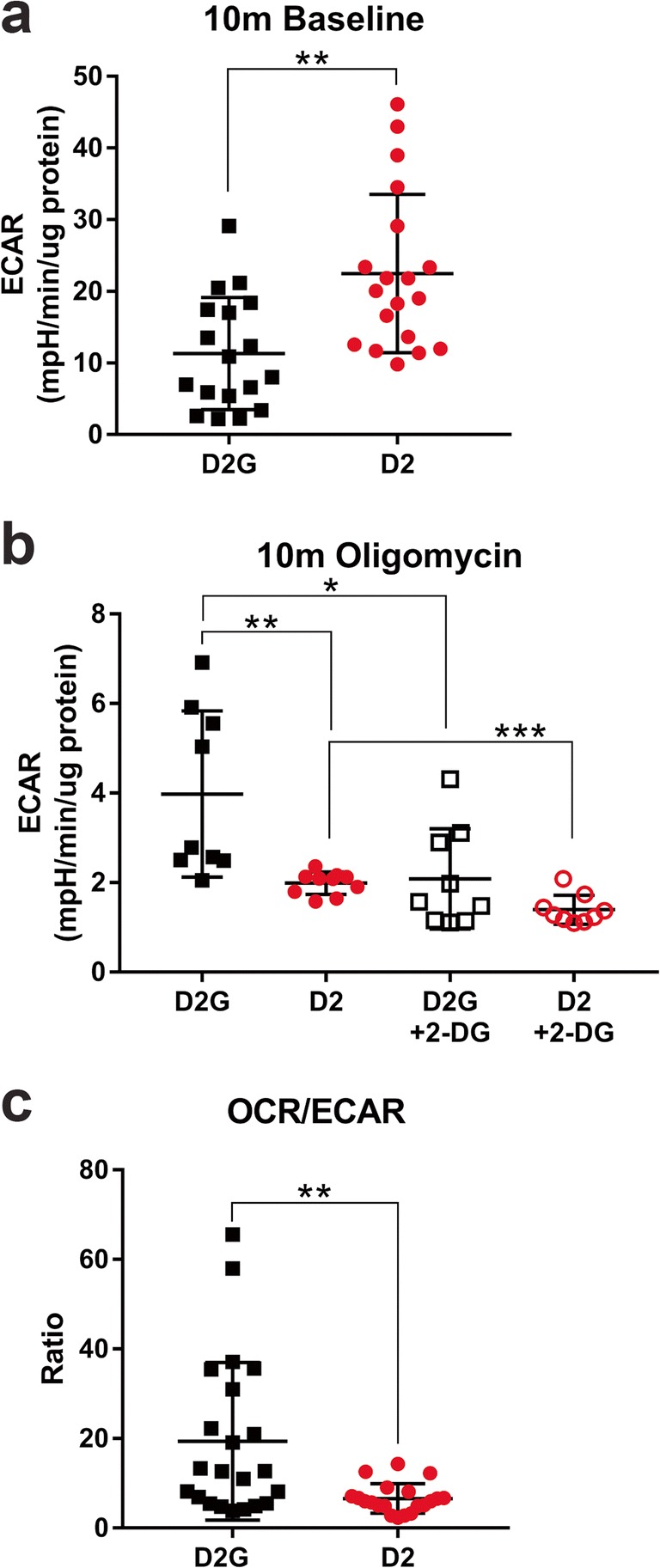
ECAR in D2 and D2G ON in the presence of FC. **a** Baseline ECAR is significantly higher in the 10-month-old D2 ON compared to the D2G, prior to FC (*t* test, **p = 0.0012). **b** ECAR with oligomycin treatment is significantly lower in the 10-month-old D2 ON compared to the D2G (*t* test, **p = 0.0011); ECAR from D2 ON with 2-deoxyglucose (2-DG) is significantly lower than D2 ON in glucose (*t* test, ***p = 0.0003); and ECAR for D2G ON with 2-DG is significantly lower than D2G ON in glucose (*t* test, **p = 0.0079). **c** The ratio of OCR to ECAR in the 10-month-old ONs shows the D2 has significantly lower ratio than the D2G (*t* test, **p = 0.0042)

The 10-month D2 ON exhibited significantly higher baseline ECAR activity (release of protons) prior to FC injection than D2G ON (Fig. 6a). ECAR in the presence of FC did not change (Online Resource Figure 3d), suggesting that the ECAR in the ON does not appreciably comprise proton release from astrocytic respiration. ECAR was not different between D2 and D2G ON at 3 months nor at 6 months during oligomycin treatment in the presence of FC (data not shown). However, at 10 months of age, ECAR was significantly greater in the D2G ON compared to the D2 ON with oligomycin treatment in the presence of FC, indicating that inhibition of the F_0_F_1_-ATPase increased glycolysis (as shown with increased ECAR) in D2G ON to meet ATP need, but the D2 ON showed an impaired ability to meet this challenge (Fig. 6b). We also measured ECAR from D2 and D2G ON that were incubated with 2-deoxyglucose (2-DG) instead of glucose. Without glucose, glycolysis should be hampered, and ECAR levels ascribable to glycolysis should also decrease. This occurred in both D2 and D2G ON, as shown in Fig. 6b. It is possible to estimate the ECAR that comes from substrate oxidation in both the D2 and D2G by comparing the difference in ECAR with and without 2-DG. In the D2 ON, the decline in ECAR ratio with glucose versus 2-DG is 29%. Therefore, ~ 71% of the ECAR ratio in the D2 ON is likely from substrate oxidation-based proton release. For the D2G ON, the ECAR ratio decreased by 50% with 2-DG compared to glucose, indicating about half of the ECAR ratio is from substrate oxidation. Collectively, these data indicate that having higher levels of ECAR in the 10-month D2 axons (Fig. 6a, b) is accompanied by an impairment in their ability to respond to quickly to changing energy conditions, especially if ATP production by oxidative phosphorylation is blocked.

The ratio of OCR to ECAR is used to show overall preference for oxidative phosphorylation or glycolysis. In Fig. 6c, the OCR to ECAR ratio is significantly higher in the D2G ON, demonstrating that the D2G ON has a much higher preference for oxidative phosphorylation than the D2 ON.

## Discussion

This study set out to determine neurodegeneration-associated changes in mitochondrial respiration and glycolysis in the ON. In the experiments without fluorocitrate, we observed decreased ATP production with progression of optic neuropathy in the D2 glaucoma model. These alterations may be a result of mitochondrial defect, or alternatively, the decreases in glucose transporter GLUT1 and monocarboxylate transporter MCT2 that occur in the 10-month-old D2 ON [3]. Interestingly, the largest decrease in ATP production in the D2 ON occurred at the 6-month time point, the same age at which MCT2, the monocarboxylate transporter specific to the RGC axon, is significantly decreased in the D2 ON. MCT2 functions to bring monocarboxylates like lactate, pyruvate, and ketone bodies into the axon. Lack of substrate availability is one contributor to decreased ATP production [29]. Another source of decreased ATP production is likely the diminished activity of electron transport chain complexes I, III, and IV that were observed as early at 5 months of age in the D2 ON [31]. Also, at 6 months of age in the D2 ON, AMPK activation is significantly increased, indicating the ON requires more energy than it is able to obtain [3]. These findings were corroborated by the lower ATP production observed in the D2 ON here.

OCR after oligomycin, FCCP, and antimycin A treatments did not vary across strain by age in the experiments run without fluorocitrate. The modest drop in OCR with oligomycin treatment for both strains suggests that mitochondrial ATP production does not contribute a significant degree to overall ATP production. Alternatively, it may indicate that glycolysis is predominant in this context. The decreased ATP production within the D2 strain, then, suggests reliance on glycolysis. Interestingly, a recently available RNA-sequencing dataset generated by the isolation of RGCs from D2 and D2G retina [10] showed upregulation of mRNA for key glycolysis enzymes in 9-month-old D2 RGCs, including hexokinase-1, aldolase A, and lactate dehydrogenase (LDH) A. We have also observed LDH-A mRNA upregulation in the 10-month D2 ON (data not shown). The mRNA data is suggestive of increased glycolysis in the D2 RGCs and their axons. Interestingly, the 10 m D2 ON has significantly decreased lactate levels as compared to the age-matched D2G (7). Low lactate in an ON that appears to be dependent upon glycolysis suggests that the lactate byproduct from glycolysis may be used as fuel by cells requiring substrate.

The average OCR after FCCP treatment exceeded the baseline OCR until 10 months of age for both the D2 and the D2G ON. This suggests an aging effect on maximal respiration. The lack of a significant increase in OCR with FCCP treatment suggests that aging and oxidative damage have impaired the electron transport chain, as corroborated by one other study [31]. Substrate supply can also impact maximal respiration, as can metabolic enzymatic rates. As mentioned above, substrate supply is likely compromised in D2 ON because of decreased GLUT1 and MCT2 [3].

Decreased spare respiratory capacity, or the ability of a cell to meet increased ATP demand, was observed in both strains with aging, but the decrease was statistically different across age for the D2 ON. Conditions of severe stress, including oxidative stress or ischemia, and decreased substrate availability can deplete the spare respiratory capacity [32]. Oxidative stress has been documented in the D2 ON [33], and ischemia may be implicated in normal tension glaucoma [34]. Higher basal OCR with decreased spare capacity is a combination previously observed in trigeminal nucleus brain sections from a model of migraine [11].

### Fluorocitrate Findings

The FC experiments endeavored to eliminate the contribution of astrocyte and oligodendrocyte mitochondria through preferential uptake of the FC by these cells and inhibition of aconitase [24], thereby providing a more sophisticated view of mitochondrial function in the axons. In the presence of FC, baseline OCR is higher in the 10-month D2 ON. The greater drop in OCR with oligomycin treatment in the D2 ON indicated more ATP production in the D2 as well. Given the increased fission [8] and greater signs of damaged/dysfunctional mitochondria in the D2 ON at this age [7, 9], it is surprising to find high OCR and greater ATP production in the D2 ON. It is possible, though, that the axonal mitochondria exhibit high OCR and ATP output because they must compensate for the dysfunctional among them. It may also be the case that pathological processes have selected for the survival of particularly efficient mitochondria. Noteworthy is that the D2 ON at 10 months is undergoing tremendous change, including reductions in axon action potential [6], mild to moderate loss of axons [5], and glial hypertrophy [35, 36]. Glial growth would increase energy demand, though these needs would not be met by axonal mitochondrial respiration. We have shown in a past study that ATP content of 10-month-old D2 ON is significantly decreased compared to 6-month D2 ON, and the higher the IOP of the animal, the lower the ATP content [6]. The Seahorse-based observations of high ATP production in 10-month-old D2 ON are nevertheless consistent with decreased overall ATP content as previously shown because (1) we are comparing 10-month-old D2 ON OCR to 10-month-old D2G without the contribution of glial respiration, and (2) we showed that OCR in the presence of oligomycin declines significantly with age in the D2. OCR decline in the presence of oligomycin is generally attributable to ATP production, and is modified by proton leak and non-mitochondrial respiration.

High oxygen consumption, as observed here for the 10-month D2 ON, can also come from non-mitochondrial respiration or proton leak. However, we determined that proton leak was not different between the glaucoma and control mice at 10 months. The 10-month D2 ON mitochondria were more highly coupled than the D2G, also suggesting that proton leak was not contributing to the higher OCR.

Lower maximal respiration was observed in the D2 ON, starting at 6 months and extending to 10 m. Since maximal respiration is primarily determined by substrate supply and oxidation, lower maximal respiration corroborates the potentially compromised substrate delivery expected in the D2 ON that has lower levels of GLUT1 and MCT2 [3], restricting substrate getting across the plasma or mitochondrial membrane [29]. The number of mitochondria and/or the cristae density can also limit maximal respiration. As shown by our research [7, 9] and others [37], mitochondria have compromised cristae and smaller size in the D2 ON. Despite the lower maximal respiration, the 10-month D2 ON nevertheless had a significantly higher ratio of ATP-linked respiration to maximal respiration. The higher ratio of ATP-linked respiration indicates that functioning mitochondria in the D2 ON axons were more efficiently producing ATP; however, since the ratio was less than 1 for both D2 and D2G (with FC) at 10 months but the D2 was significantly higher, the D2 ON is working closer to its maximum output capacity than the D2G. The high oxygen consumption for ATP production in the D2 may exist to compensate for the glaucoma-related changes in the ON that contribute to energy depletion, including compromised access to lactate and increased levels of activated AMPK [3].

The cut ends of axons, created by chopping the ON into pieces for the assay, could be exposed to fluorocitrate, potentially allowing the compound to interfere with axonal oxidative phosphorylation. OCR fell by ~ 50 pmol/min/μg protein with the addition of fluorocitrate, a level that represented between 25 and 30% of the baseline OCR, suggesting that its impact on OCR is consistent with expectations for blocking astrocyte, but little of the axonal, OCR.

### ECAR Analysis

Extracellular acidification rate (ECAR) at baseline was significantly higher in the 6- and 10-month D2 compared to the D2G ON (without FC). Glycolysis upregulation, evident in both strains by the positive difference in ECAR with oligomycin treatment over baseline, may compensate for failing mitochondria in aged D2 and D2G ON. The high ECAR values in the D2 ON suggest higher levels of glycolysis in D2 than D2G ON. We had determined that GLUT1 levels were significantly decreased in 10-month-old D2 ON; higher glycolysis may account for the significantly lower levels of glycogen also observed in 10-month-old D2 ON [3] if those pools are being utilized at the expense of incoming substrate. However, as discussed below, glycolysis appears to comprise a smaller fraction than expected of the ECAR output in D2 ON.

In the presence of FC, ECAR at baseline was significantly higher in the 10-month D2 ON than the D2G. An initial interpretation of such an observation would be significantly higher glycolysis in the 10-month D2 ON. However, ECAR represents proton release from glycolysis as well as TCA cycle substrate oxidation. The ratio of one to the other varies by cell type and context [30]. It was only in the fluorocitrate experiments that we were able to estimate the contribution of respiration to ECAR. Roughly 30% of the D2 ON ECAR fell when 2-DG was the substrate in the media instead of glucose, suggesting that 70% of the ECAR under these conditions was derived from the HCO_3_^−^ + H^+^ given off by TCA cycle CO_2_. These figures likely overestimate the contribution of substrate oxidation because glycolysis was not completely inhibited with 2-DG in this context due to the residual glucose in the media. In addition, endogenous sources of glucose, such as glycogen, can serve as a source for glucose in the presence of 2-DG and thus, contribute to ECAR. This may explain why, in contrast to the D2 ON, ECAR in the D2G ON was evenly split (50-50) between glycolysis and respiration. D2G ON maintains significantly higher glycogen stores than D2 ON, for which stores are depleted with glaucoma pathogenesis [3]. These estimates of the source of ECAR assists our interpretation of the ECAR data. For the FC experiments, glial respiration was constrained. The lack of change in ECAR between baseline and FC treatment indicated that glial respiration did not contribute in an appreciable way to ECAR levels. On one level, this meets expectations of glia as glycolytic cells [38]. However, astrocyte mitochondria comprise the majority of mitochondrial mass in the ON [39], providing astrocytes with significant capacity for oxidative phosphorylation. In one analysis of glucose versus lactate substrate utilization in brain cortex, astrocytes oxidized 50% of the interstitial lactate and 35% of the glucose, while neurons oxidized no more than 50% of the lactate and 65% of the glucose found in the parenchyma [27]. These findings imply that cortical astrocytes preferentially utilize oxidative phosphorylation. Despite significant mitochondrial mass in the ON, these data suggest glial cell mitochondria nevertheless do not appear to contribute significantly to ECAR through substrate oxidation.

Axonal mitochondria respiration comprises a major portion of the ECAR, but this is likely context dependent. It was only in the fluorocitrate experiments that we were able to estimate ECAR contribution from glycolysis versus substrate oxidation. The D2 ON axonal mitochondria at 10 months of age were exceptionally efficient and productive compared to the D2G ON mitochondria. Oxidizing substrate would have increased ECAR, and did so to a larger degree in D2 than D2G. It is not surprising, then, that ~ 70% of D2 ON ECAR could be attributable to axonal mitochondria respiration. D2 ON residual functional mitochondria have to compensate for degenerative changes and an apparent glycolysis limit. An additional piece of corroborating evidence is the significant decline in lactate levels in the 10-m D2 ON compared to D2G (7); low lactate either indicates that glycolysis is decreased, or that the lactate is being utilized as fuel in the D2 ON more so than in the D2G.

An important finding for the metabolic contribution to D2 optic neuropathy is that oligomycin in the presence of FC did not increase ECAR to the degree that it did in the D2G ON. The ECAR boost in the D2G ON with oligomycin treatment was double the ECAR observed in the D2 ON at 10 months of age. This indicates that the D2 ON was not capable of boosting glycolysis to meet ATP demand as well as the D2G ON. When challenged with ATPase inhibition, D2G switches to glycolysis; but the 10-month D2 ON was not as capable at glycolysis upregulation. Since we have indications that ECAR, for the D2 ON, is primarily from axonal mitochondria respiration, these data corroborate the limited ability of the D2 ON at 10 months to increase respiration with FCCP treatment. This lack of a true boost to glycolysis in the D2 ON with oligomycin could be a result of glucose uptake challenges inherent in an ON in which the GLUT1 protein levels were significantly decreased [3], or an indication of some constraint on glycolysis, such as inhibition of a rate-limiting enzyme or saturation of glycolytic flux. Phosphofructokinase, a rate-limiting enzyme in glycolysis, can be inhibited by citrate, a TCA cycle intermediate, as well as ATP. If oxidative phosphorylation is proceeding as well as indicated in the D2 ON with FC, then it is possible that glycolysis would be inhibited. However, that inhibition should have been relieved by oligomycin treatment. As previously discussed, mRNA for a number of important glycolytic enzymes are significantly upregulated in the D2 RGCs compared to either younger D2 or RGCs taken from D2G mice [10]. Further analysis of enzyme protein levels and activities will enable greater insight into the balance of metabolism in the degenerating D2 visual system.

### RGC and Visual Function Indices Do Not Predict Metabolic Changes

As shown in the scatterplots of RGC density or visual acuity by respiration, mice with fewer RGCs or lower visual acuity did not demonstrate significantly greater deficits in OCR than other mice within their group. This result is attributable to many things, including the mixed population of cells and axons in the ON that can obscure bioenergetic analysis, the presence of degenerating axons in the D2 10-month-old samples, and the high variability of OCR data. It is important to remember that OCR is a readout of mitochondrial function, not mitochondrial number or dynamics. However, ON populations did reveal significant differences of metabolic management between the glaucomatous and control ON, including an inability to metabolically switch to glycolysis when called upon, and an untenable reliance on oxidative phosphorylation.

## Electronic Supplementary Material

Supplementary Fig. 1 **a.** Scatterplot of maximal respiration by visual acuity in 10 month-old D2 and D2G mice. Linear regression of D2 maximal respiration and visual acuity had an R-squared value of 0.1259 (F_1,33_ = 4.752, *p* = 0.0365), indicating that 12% of the variance in maximal respiration could be ascribed to visual acuity. The slope of the linear regression for the D2G maximal respiration by visual acuity was not different from zero. **b.** Scatterplot of normalized basal respiration by RGC density in 10 month-old D2 and D2G mice. The range of RGC density is wider in the D2 mouse, and the regression line in the D2 has no slope; there is a slight upward slope for the D2G mice, though they are not statistically different. (PNG 172 kb)

High Resolution (TIF 1364 kb)

Supplementary Fig. 2 **a.** ATP production values, as calculated from baseline normalized data, in ON grouped by visual acuity for D2 and D2G mice at 10 months of age. Values overlap across all visual acuities and strains. Visual acuity was binned by spatial frequency of 0.1 cycles/degree. There were no mice with visual acuity lower than 0.2 cyc/deg. in the D2G group. **b.** Maximal respiration values in ON grouped by visual acuity for D2 and D2G mice at 10 months of age. For the D2 ON, maximal respiration shows a trend toward increasing values with higher visual acuity, but the results are not statistically significant. There are no differences in maximal respiration for the three visual acuity bins for the D2G mouse. **c.** Baseline extracellular acidification rate (ECAR) grouped by visual acuity for the D2 and D2G mice at 10 months of age. At higher visual acuity (0.3 cyc/deg. and above), the D2 and D2G strains are not statistically different. D2 mice with poor visual acuity drive the significantly higher overall baseline ECAR as shown in Fig. 4. (PNG 278 kb)

High Resolution (TIF 2159 kb)

Supplementary Fig. 3 Oxygen consumption rate normalized to protein per well for the 10 month D2 (**a**) and D2G (**b**) ON in the fluorocitrate (FC) experiments. The difference in OCR from baseline to FC treatment corresponds to the oxygen consumption attributable to glial mitochondria whose aconitase is specifically inhibited by FC. **c.** Isolated bovine mitochondria were incubated with or without 2500 μM fluorocitrate for 30 min, then aconitase activity was measured in a plate-reader based assay (Cayman Chemical, 705,502). **d.** ECAR is significantly higher in the 10 month-old D2 ON compared to the D2G in the presence of FC (t-test, **p = 0.0016). (PNG 285 kb)

High Resolution (TIF 1654 kb)

Supplementary Fig. 4 Extracellular acidification rate (ECAR) for 3 month (a, c, e) and 6 month (b, d, f) groups at each stage of the mitochondrial stress test in the fluorocitrate experiments. Panels **a** and **b** show baseline ECAR for the D2 and D2G mouse optic nerve. Panels **c** and **d** show the ECAR after addition of fluorocitrate. There is no difference across D2 and D2G groups, no differences across 3 and 6 month age groups, and no difference between ECAR at baseline and with fluorocitrate treatment (compare **a** and **b** to **c** and **d**, respectively). In panels **e** and **f**, with oligomycin addition, wells also received either glucose (D2G and D2) or 2-deoxyglucose (D2G + 2-DG and D2 + 2-DG). Note the general increase in ECAR from 3 to 6 months with oligomycin addition; this apparent increase is not statistically different. ECAR with 2-deoxyglucose addition is generally lower than with glucose at both 3 (panel **e**) and 6 (panel **f**) months, reflecting the decrease in lactate and proton production when glycolysis is halted. The values do not go to zero because of residual glucose (2 mM) in the media. (PNG 235 kb)

High Resolution (TIF 1879 kb)

## Abbreviations

AA: antimycin A
AD: Alzheimer’s disease
AMPK: adenosine monophosphate activated protein kinase
ATP: adenosine triphosphate
CNS: central nervous system
CTB: cholera toxin-B subunit
D2: DBA/2J mouse
D2G: DBA/2J-*Gpnmb*^+^
DMEM: Dulbecco’s modified essential medium
ECAR: extracellular acidification rate
FC: fluorocitrate
FCCP: carbonyl cyanide 4-(trifluoromethoxy)phenylhydrazone
GLUT1: glucose transporter 1
IOP: intraocular pressure
MCT2: monocarboxylate transporter 2
OCR: oxygen consumption rate
ON: optic nerve
PBS: phosphate buffered saline
RBPMS: RNA binding protein, multiple splicing
RGC: retinal ganglion cell
TCA: tricarboxylic acid.
