# Supplementary figures and images for: Higher Reliance on Glycolysis Limits Glycolytic Responsiveness in Degenerating Glaucomatous Optic Nerve
Source: Mol Neurobiol. 2019 Apr 13;56(10):7097–112. doi: 10.1007/s12035-019-1576-4 (PMC6728180; doi:10.1007/s12035-019-1576-4)

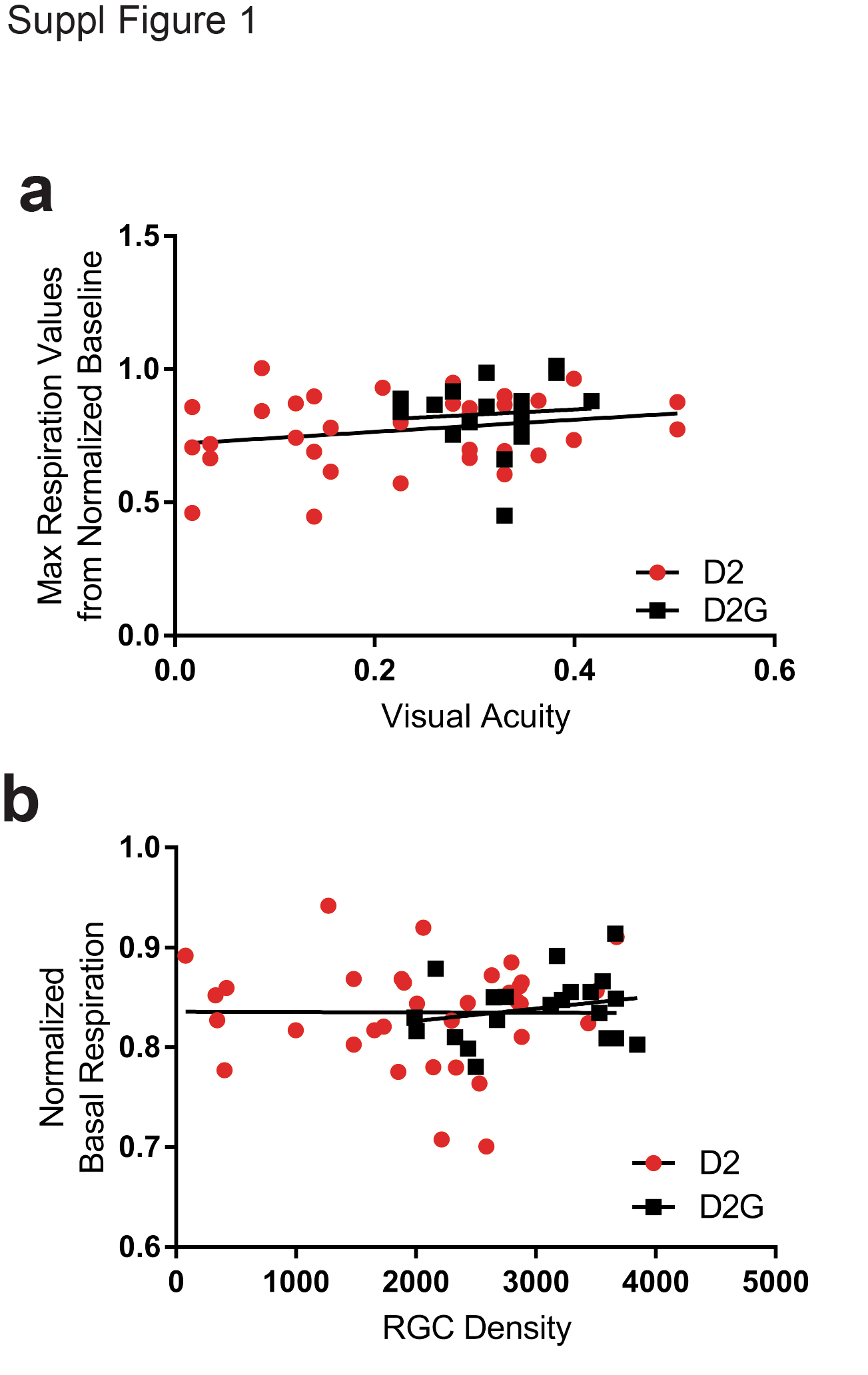

Supplement: Supplementary file 1 — a. Scatterplot of maximal respiration by visual acuity in 10 month-old D2 and D2G mice. Linear regression of D2 maximal respiration and visual acuity had an R-squared value of 0.1259 (F1,33 = 4.752, p = 0.0365), indicating that 12% of the variance in maximal respiration could be ascribed to visual acuity. The slope of the linear regression for the D2G maximal respiration by visual acuity was not different from zero. b. Scatterplot of normalized basal respiration by RGC density in 10 month-old D2 and D2G mice. The range of RGC density is wider in the D2 mouse, and the regression line in the D2 has no slope; there is a slight upward slope for the D2G mice, though they are not statistically different. (PNG 172 kb) [file 12035_2019_1576_Fig7_ESM.png]

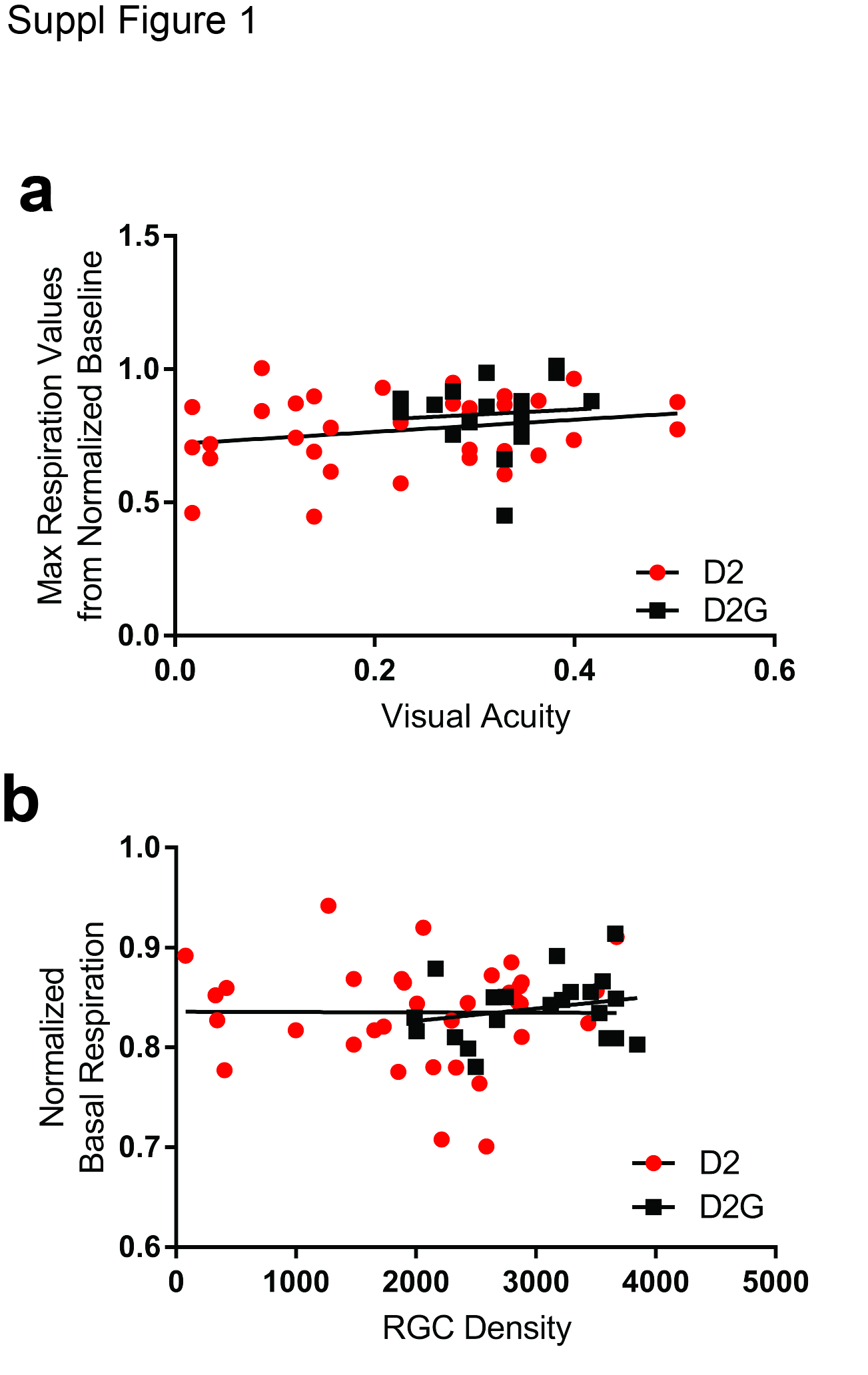

Supplement: Supplementary file 2 — High Resolution (TIF 1364 kb) [file 12035_2019_1576_MOESM1_ESM.tif]

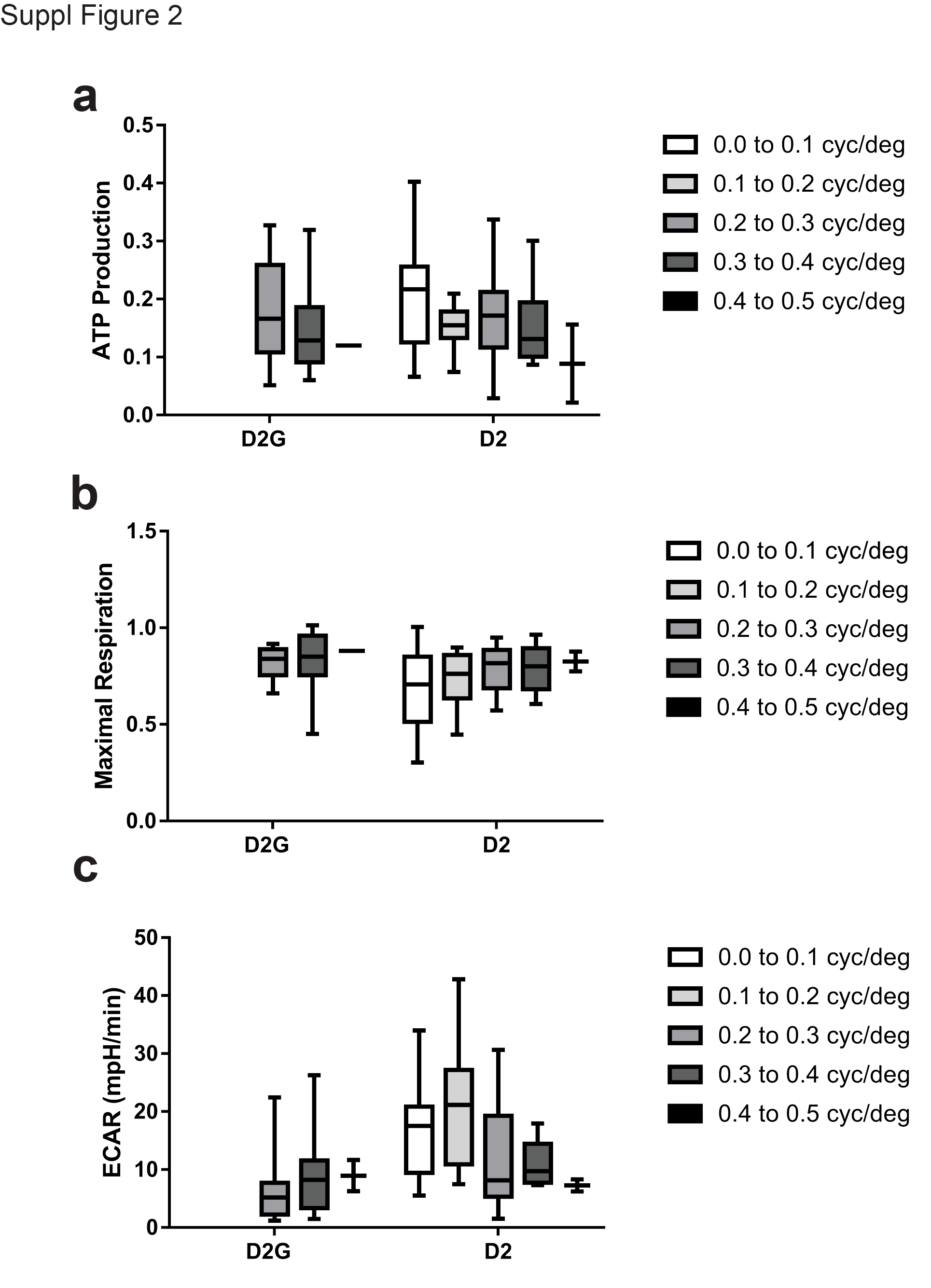

Supplement: Supplementary file 3 — a. ATP production values, as calculated from baseline normalized data, in ON grouped by visual acuity for D2 and D2G mice at 10 months of age. Values overlap across all visual acuities and strains. Visual acuity was binned by spatial frequency of 0.1 cycles/degree. There were no mice with visual acuity lower than 0.2 cyc/deg. in the D2G group. b. Maximal respiration values in ON grouped by visual acuity for D2 and D2G mice at 10 months of age. For the D2 ON, maximal respiration shows a trend toward increasing values with higher visual acuity, but the results are not statistically significant. There are no differences in maximal respiration for the three visual acuity bins for the D2G mouse. c. Baseline extracellular acidification rate (ECAR) grouped by visual acuity for the D2 and D2G mice at 10 months of age. At higher visual acuity (0.3 cyc/deg. and above), the D2 and D2G strains are not statistically different. D2 mice with poor visual acuity drive the significantly higher overall baseline ECAR as shown in Fig. 4. (PNG 278 kb) [file 12035_2019_1576_Fig8_ESM.png]

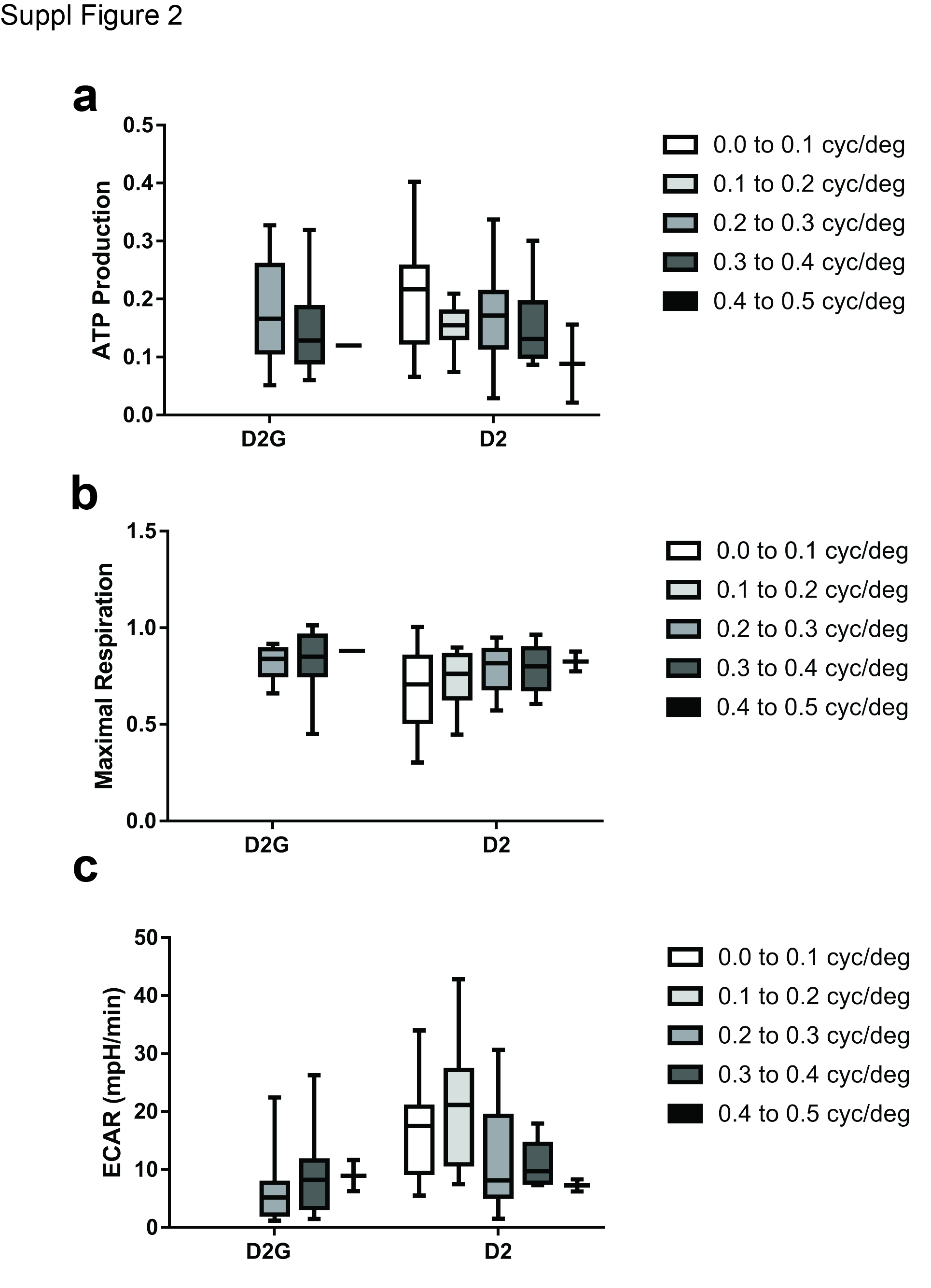

Supplement: Supplementary file 4 — High Resolution (TIF 2159 kb) [file 12035_2019_1576_MOESM2_ESM.tif]

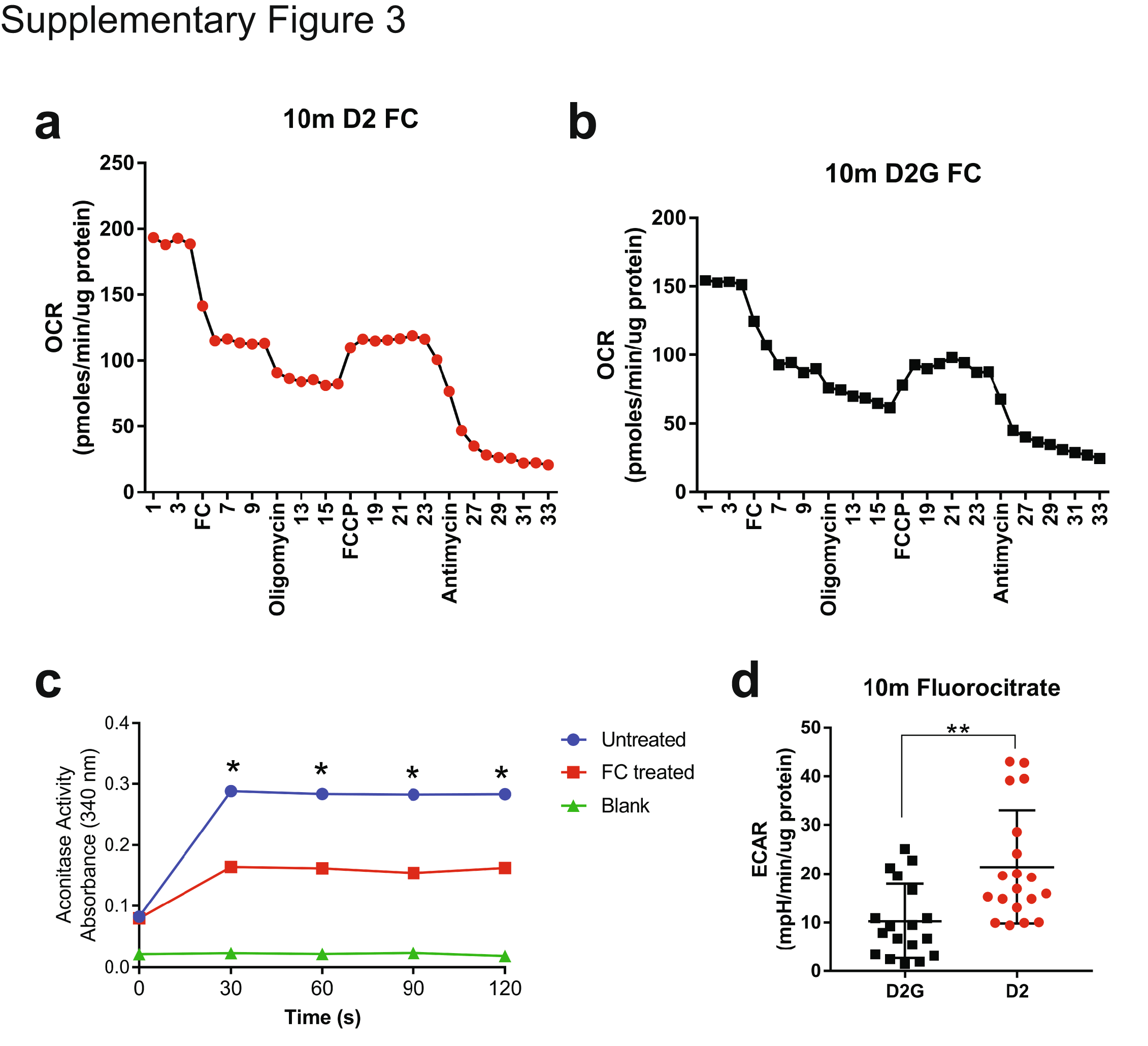

Supplement: Supplementary file 5 — Oxygen consumption rate normalized to protein per well for the 10 month D2 (a) and D2G (b) ON in the fluorocitrate (FC) experiments. The difference in OCR from baseline to FC treatment corresponds to the oxygen consumption attributable to glial mitochondria whose aconitase is specifically inhibited by FC. c. Isolated bovine mitochondria were incubated with or without 2500 μM fluorocitrate for 30 min, then aconitase activity was measured in a plate-reader based assay (Cayman Chemical, 705,502). d. ECAR is significantly higher in the 10 month-old D2 ON compared to the D2G in the presence of FC (t-test, **p = 0.0016). (PNG 285 kb) [file 12035_2019_1576_Fig9_ESM.png]

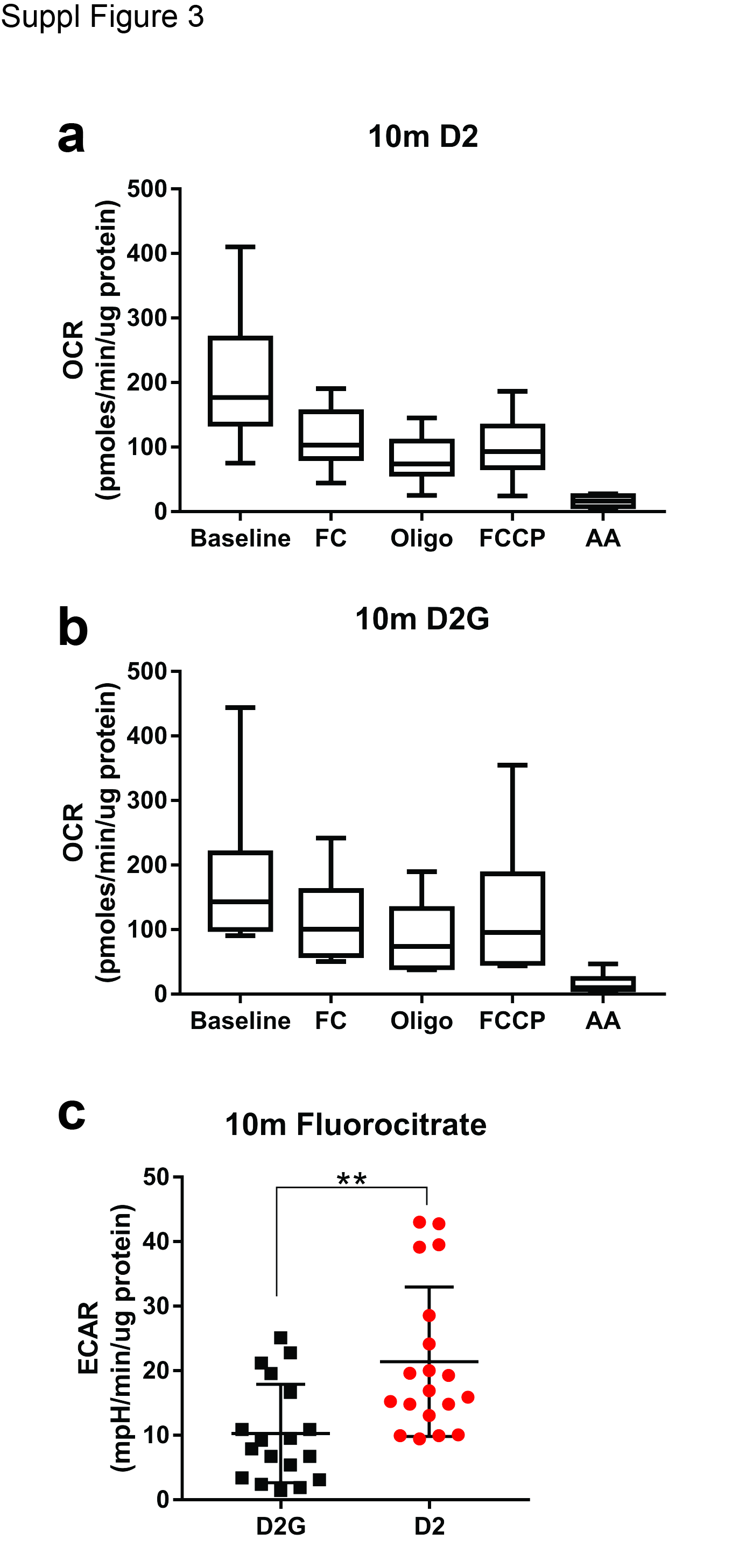

Supplement: Supplementary file 6 — High Resolution (TIF 1654 kb) [file 12035_2019_1576_MOESM3_ESM.tif]

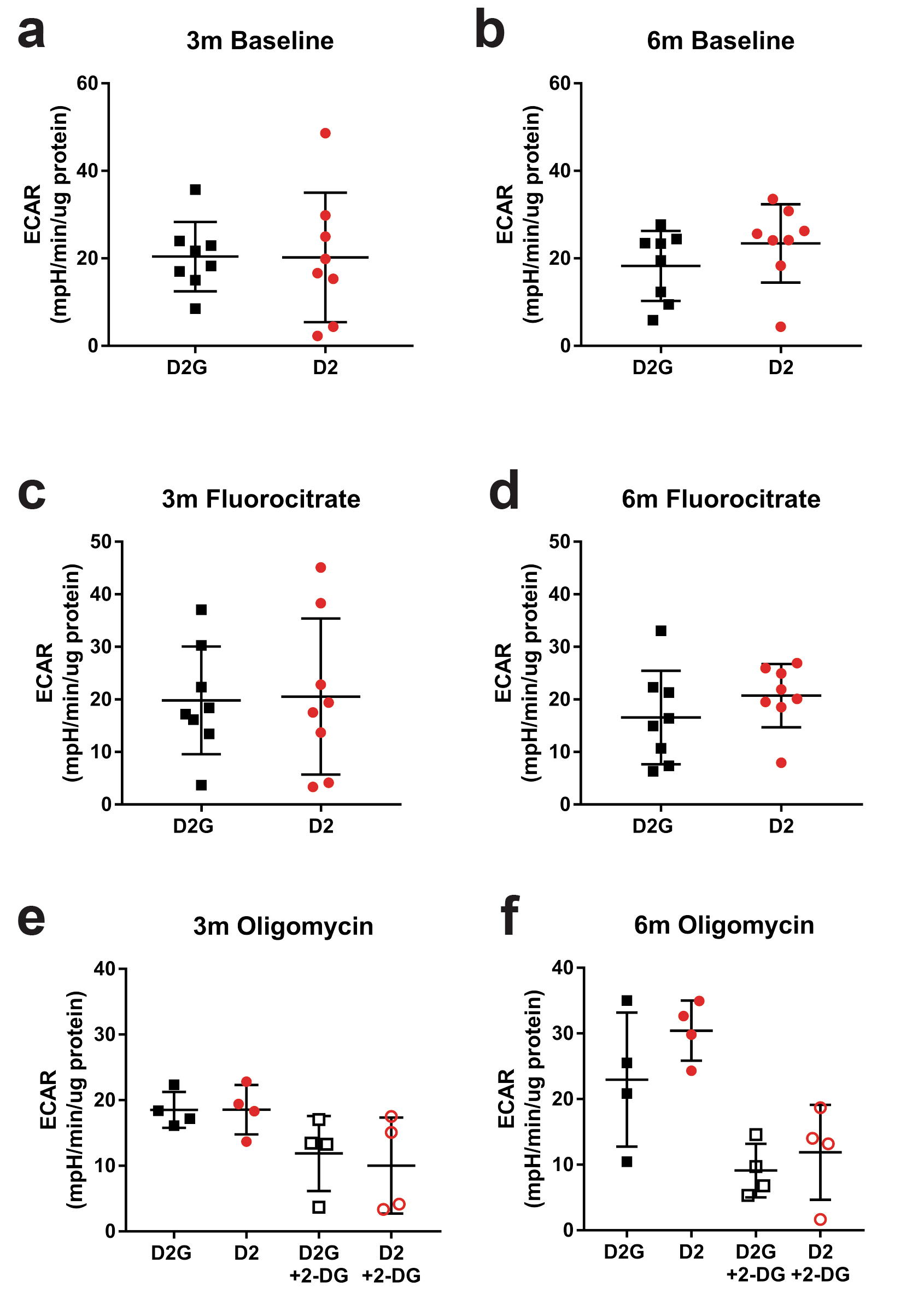

Supplement: Supplementary file 7 — Extracellular acidification rate (ECAR) for 3 month (a, c, e) and 6 month (b, d, f) groups at each stage of the mitochondrial stress test in the fluorocitrate experiments. Panels a and b show baseline ECAR for the D2 and D2G mouse optic nerve. Panels c and d show the ECAR after addition of fluorocitrate. There is no difference across D2 and D2G groups, no differences across 3 and 6 month age groups, and no difference between ECAR at baseline and with fluorocitrate treatment (compare a and b to c and d, respectively). In panels e and f, with oligomycin addition, wells also received either glucose (D2G and D2) or 2-deoxyglucose (D2G + 2-DG and D2 + 2-DG). Note the general increase in ECAR from 3 to 6 months with oligomycin addition; this apparent increase is not statistically different. ECAR with 2-deoxyglucose addition is generally lower than with glucose at both 3 (panel e) and 6 (panel f) months, reflecting the decrease in lactate and proton production when glycolysis is halted. The values do not go to zero because of residual glucose (2 mM) in the media. (PNG 235 kb) [file 12035_2019_1576_Fig10_ESM.png]

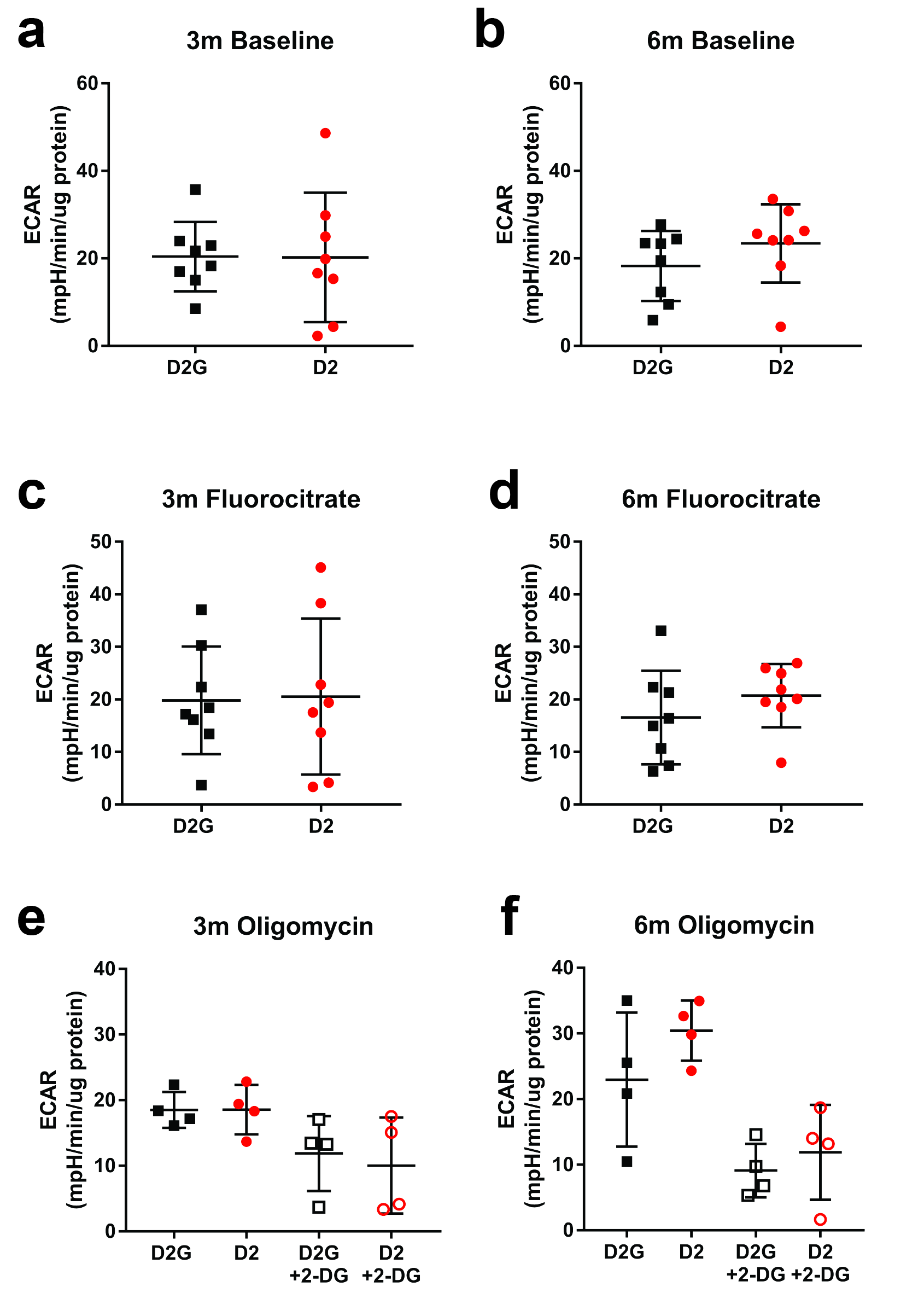

Supplement: Supplementary file 8 — High Resolution (TIF 1879 kb) [file 12035_2019_1576_MOESM4_ESM.tif]
